# Oral Pathobiont Activates Anti-Apoptotic Pathway, Promoting both Immune Suppression and Oncogenic Cell Proliferation

**DOI:** 10.1038/s41598-018-35126-8

**Published:** 2018-11-09

**Authors:** Pachiappan Arjunan, Mohamed M. Meghil, Wenhu Pi, Jinxian Xu, Liwei Lang, Ahmed El-Awady, William Sullivan, Mythilypriya Rajendran, Mariana Sousa Rabelo, Tong Wang, Omnia K. Tawfik, Govindarajan Kunde-Ramamoorthy, Nagendra Singh, Thangaraju Muthusamy, Cristiano Susin, Yong Teng, Roger M. Arce, Christopher W. Cutler

**Affiliations:** 1 Department of Periodontics, Dental College of Georgia, Augusta University, Augusta, Georgia United States of America; 2 Department of Radiation Oncology, Indiana University, Indianapolis, Indiana United States of America; 3 Department of Oral Biology, Augusta University, Augusta, Georgia United States of America; 4 Department of Energy, Joint Genome Institute, California, United States of America; 5 Department of Periodontics, University of São Paulo, Sao Paulo, Brazil; 6 The Jackson Laboratory for Genomic Medicine, Connecticut, United States of America; 7 Department of Biochemistry & Molecular Biology, Cancer Research Center, Augusta University, Augusta, Georgia United States of America

## Abstract

Chronic periodontitis (CP) is a microbial dysbiotic disease linked to increased risk of oral squamous cell carcinomas (OSCCs). To address the underlying mechanisms, mouse and human cell infection models and human biopsy samples were employed. We show that the ‘keystone’ pathogen *Porphyromonas gingivalis*, disrupts immune surveillance by generating myeloid-derived dendritic suppressor cells (MDDSCs) from monocytes. MDDSCs inhibit CTLs and induce FOXP3 + T_regs_ through an anti-apoptotic pathway. This pathway, involving pAKT1, pFOXO1, FOXP3, IDO1 and BIM, is activated in humans with CP and in mice orally infected with Mfa1 expressing *P. gingivalis* strains. Mechanistically, activation of this pathway, demonstrating FOXP3 as a direct FOXO1-target gene, was demonstrated by ChIP-assay in human CP gingiva. Expression of oncogenic but not tumor suppressor markers is consistent with tumor cell proliferation demonstrated in OSCC-*P. gingivalis* cocultures. Importantly, FimA + *P. gingivalis* strain MFI invades OSCCs, inducing inflammatory/angiogenic/oncogenic proteins stimulating OSCCs proliferation through CXCR4. Inhibition of CXCR4 abolished *Pg*-MFI-induced OSCCs proliferation and reduced expression of oncogenic proteins SDF-1/CXCR4, plus pAKT1-pFOXO1. Conclusively, *P. gingivalis*, through Mfa1 and FimA fimbriae, promotes immunosuppression and oncogenic cell proliferation, respectively, through a two-hit receptor-ligand process involving DC-SIGN^+hi^/CXCR4^+hi^, activating a pAKT^+hi^pFOXO1^+hi^BIM^−low^FOXP3^+hi^ and IDO^+hi^- driven pathway, likely to impact the prognosis of oral cancers in patients with periodontitis.

## Introduction

The Centers for Disease Control and Prevention indicates that in the U.S. nearly 50% of adults have moderate periodontitis, while the World Health Organization predicts 10–15% of adults are affected by advanced periodontitis^1^. Chronic periodontitis (CP) is linked to increased risk of many systemic diseases^2^ including cancers of gastrointestinal (GI) tract^3^ and oral squamous cell carcinoma (OSCC)^4^. CP patients are at substantial risk for developing deadly oral cancers, 90% of which are recurrent OSCC having less than 50% survival rate^5,6^. It has been denoted that patients with CP are more susceptible to poorly differentiated OSCC than those without periodontitis, thus indicating the association of periodontal disease with the development as well as advancement of oral cancers^7,8^. A recent cohort study emphasizes the role of periodontal disease in incident cancer risk among postmenopausal women^9^. Chronic inflammation of the periodontal tissues is mainly caused by a complex interaction of oral pathogens in the dental biofilm. *Porphyromonas gingivalis* (*Pg*)^10^ is a highly influential member of the oral biofilm, causing a mucosal dysbiosis and disrupting immune homeostasis^11^ through unclear mechanisms. Although, chronic inflammation is generally regarded as a causal factor in carcinogenesis^12^, the mechanistic role of CP and its dysbiotic microflora^13,14^ in cancer risk are only now beginning to be explored^15^.

Dendritic cells (DCs) are professional antigen presenting cells that bridge the gap between innate and adaptive immunity^16^. On the contrary, DCs can also play a role in inducing immune suppression under specific circumstances. From this perspective, DCs promote tolerance rather than immunity^17^. The functional diversity and plasticity of DCs has stimulated much interest in their use for immunotherapy for cancers, autoimmune and infectious diseases^18–20^. Distinct from DCs are myeloid-derived suppressor cells (MDSCs) with at least six subsets discovered so far. MDSCs in melanoma patients are Stat3^high^ and overexpress CD80, CD83 and CD209 or Dendritic Cell-Specific Intercellular adhesion molecule-3-Grabbing Non-integrin (DC-SIGN)^21^. MDSC induction of regulatory T cells (T_regs_) contributes to tumor progression, failure of DC-based immunotherapy^22^ and murine oral cancers^23^_._ A main factor in T_reg_ induction by DCs and possibly MDSCs^24^ is their production of immunoregulatory Indoleamine 2, 3-dioxygenase (IDO) enzyme. IDO is critical for T_reg_ function and maintenance^25,26^ promoting a tolerogenic state and inducing direct T cell suppression and enhancement of local T_regs_^27^. T_regs_ control periodontitis in mice^28^, but are also reported to promote disease progression and pathogen immune escape^29,30^.

The induction of apoptosis in host cells and in their prompt removal by phagocytes is important for maintaining tissue homeostasis. This is particularly important for the development of an immunogenic response and antitumor immunity. Inhibition of apoptosis is mediated by downstream effectors of serine-threonine protein kinase- (Akt1) and Bcl-2 family members^31^. This anti-apoptotic pathway is activated in DCs by many factors including ligation of DC-SIGN^32^. DC-SIGN is a key uptake signaling receptor and immune-modulator for many pathogens, including *Mycobacterium tuberculosis*, *Helicobacter pylori*^33^, and as we have shown, fimbriated (Mfa1+) *P. gingivalis* strains^34^. Oral colonization with *P. gingivalis* leads to invasion of DCs and of their myeloid progenitors through the action of DC-SIGN ligand Mfa1 and TLR2/C-X-C chemokine receptor type 4 (CXCR4) ligand FimA. The chemokine stromal cell-derived factor 1 (SDF-1) and CXCR4 are significant markers of poor prognosis in many hematological malignancies^35^. Once phosphorylation occurs through Akt-1, the Forkhead box class-O (FOXO) proteins migrate from the nucleus and remain transcriptionally inactive resulting in their degradation or sequestration^35,36^. Since genes encoding pro-apoptotic molecules particularly Bcl-2 member Bim^37^ are activated by FOXO members, its inactivation by DC-SIGN ligation can disrupt immune homeostasis. Deletion of FOXO1^36,38^ reduces DC functions and enhances susceptibility to periodontitis in a mouse model^39^. It was described that FOXO1 silencing enhances cell proliferation and decreases apoptosis of papillary thyroid carcinoma cells via Akt-FOXO1 signaling^40^. However, the roles of phospho-Akt1 (pAKT1) in direct regulation of FOXO1 in CP or oral squamous carcinoma cells have not been described. Recently, we reported that human monocyte-derived DCs (MoDCs) exposed to *P. gingivalis* promote FOXO1 gene expression^41^. However, the mechanistic role of FOXO1/pFOXO1 in regulating myeloid cell plasticity and immune homeostasis in response to this pathogen is unknown.

We show here through a combination of human, mouse and *in-vitro* studies how the dysbiotic pathogen *P. gingivalis* disrupts immune surveillance in periodontitis. Mfa1-fimbriae expressing *P. gingivalis* strains invade monocytes and promote differentiation to apoptosis resistant IDO-competent MDDSCs. These MDDSCs induce immune tolerance through increased FOXP3 + T_reg_ responses. Moreover, our data show in inflamed periodontal tissues that FOXP3 is a direct target of pFOXO1, which is regulated by pAKT1. Combined with our evidence for direct induction of OSCCs proliferation by *P. gingivalis*, we conclude that blocking this pathway is a promising interventional approach to restore immune-surveillance and reduce oncogenic cell proliferation in dysbiotic conditions such as periodontitis.

## Results

### Transcriptome and phenotype profiling of a novel *P. gingivalis* induced myeloid subset

We have previously described the ability of *P. gingivalis* to infect monocytes isolated from human PBMCs and induce their differentiation into a novel immunoregulatory myeloid cell type^42^, that promotes T_regs_ and inhibits cytotoxic T cells^43^. While this myeloid cell type functionally resembles myeloid-derived suppressor cells (MDSCs), which have been associated with oncogenesis^22,43^, these are phenotypically a distinct subtype of immature DCs (CD14^low^CD83^−^CD1c^+^DC-SIGN^+^) which we provisionally call Myeloid Derived Dendritic Suppressor Cells (MDDSCs). Transcriptional profiling of MDDSCs reveal MDSC-mediators of immunosuppression CD15, signal transducer and activator of transcription-3 (STAT-3) and arginase-1 (ARG1), but not canonical MDSC markers CD11b, CD33, CD14 and CD16 (Fig. S1A). Flow cytometry analysis (Fig. S1B) also confirms lack of canonical MDSC markers CD16, CD33 or CD11b and HLA-DR^high^ expression. MDDSCs were subjected to further characterization by RNAseq, TaqMan qPCR and proteomics analysis; the latter to identify downstream signaling pathways. In these series of experiments, a panel of highly characterized Mfa1/FimA fimbriae deficient mutants that target distinct pattern recognition receptors (PRRs) on DCs (Table S1), as we have reported were used^34,41,44–46^, along with WT-*P. gingivalis*381 (*Pg*381), to infect monocytes, as well as canonical GM-CSF/IL-4 induced MoDCs. The RNA-sequencing analysis shows functional clustergram partitioned into 5 clusters such as 1. Angiogenic-/Oncogenic & Inflammatory markers, 2. Immunosuppression/regulation, 3. Transcription Factors, 4. Anti-apoptosis/Cell Survival, and 5. Pro-apoptosis/Cell Death in response to *Pg*381 and DPG3 compared with uninfected control. *P. gingivalis381*, which expresses both Mfa1 and FimA, induced T-cell suppression factors programmed death-ligand-1 (PD-L1 or CD274), IL-10, adenosine A2b receptor (ADORA2B), ICOS Ligand (CD275) involved in recruitment of T-cells into oral mucosal tissues, and IDO1, CD80 involved in immunosuppressive activities of MDSCs compared with uninfected control MoDCs. Also noted were transcripts related to cell growth/survival regulation (STAT3, STAT5B, and FOXO1) and angiogenic/oncogenic metastasis markers [vascular endothelial growth factor (VEGF)-A and CXCR4] (Fig. S2A). Intriguingly, DPG3, the fimbriae mutant that solely expresses Mfa1 fimbriae, and that targets DC-SIGN for uptake^46^ and survival through autophagy evasion in DCs^44^ induced the greatest number of distinct genes (Fig. S2A). Preferentially upregulated were angiogenesis-/oncogenic-/inflammation related [VEGFA, CXCR4, AKT1, adrenomedullin (ADM), interleukin 6 (IL-6) and interleukin 8 (IL-8)], immunosuppressive [IDO1, CD80, IL-10, B7 homolog1 or Programmed death-ligand 1 (B7-H1 or PD-L1/CD274)] and transcription/anti-apoptosis/DC proliferation related (STAT3, STAT5B, FOXO1 and AKT1) genes, while pro-apoptotic (BIM, BAD and CASP8)- genes were downregulated. In addition, it’s worth noting that the cell proliferation and oncogenic gene AKT1, angiogenic molecule VEGF-A, immunosuppressive molecule IDO1, IL-10, the transcription factor STAT3 were highly upregulated (≥20 FPKM), whereas pro-apoptotic/cell death related genes BIM, BAD, CASP8 were down-regulated in DPG3 compared with *Pg*381. Quantitative PCR data further confirms that DPG3 considerably increased (>10-fold) the expression of CD274, IDO1, AKT1, VEGF, CXCR4, STAT5B, FOXO1, and survivin or baculoviral inhibitor of apoptosis repeat-containing 5 (BIRC5) genes (Fig. S2B) compared with uninfected control. In contrast pro-apoptotic BIM, CASP8 and tumor suppressors FOXO3 and LDOC1 related genes were (≥5 folds) decreased, significantly, compared with *Pg381* and uninfected control (Fig. S2A, B). Besides, immunoblot shows the decreased expression of BIM at protein level in DPG3 induced MDDSCs compared with *Pg381*, MFI and uninfected control MoDCs (Fig. S2C). These data collectively delineate that DPG3 was the most potent inducer of these MDDSCs.

### *P. gingivalis* expressing Mfa1 (DPG3) directs induction of anti-apoptotic and pAKT1-pFOXO1 mediated oncogenic signaling pathway in MDDSCs through DC-SIGN

**Figure 1:**
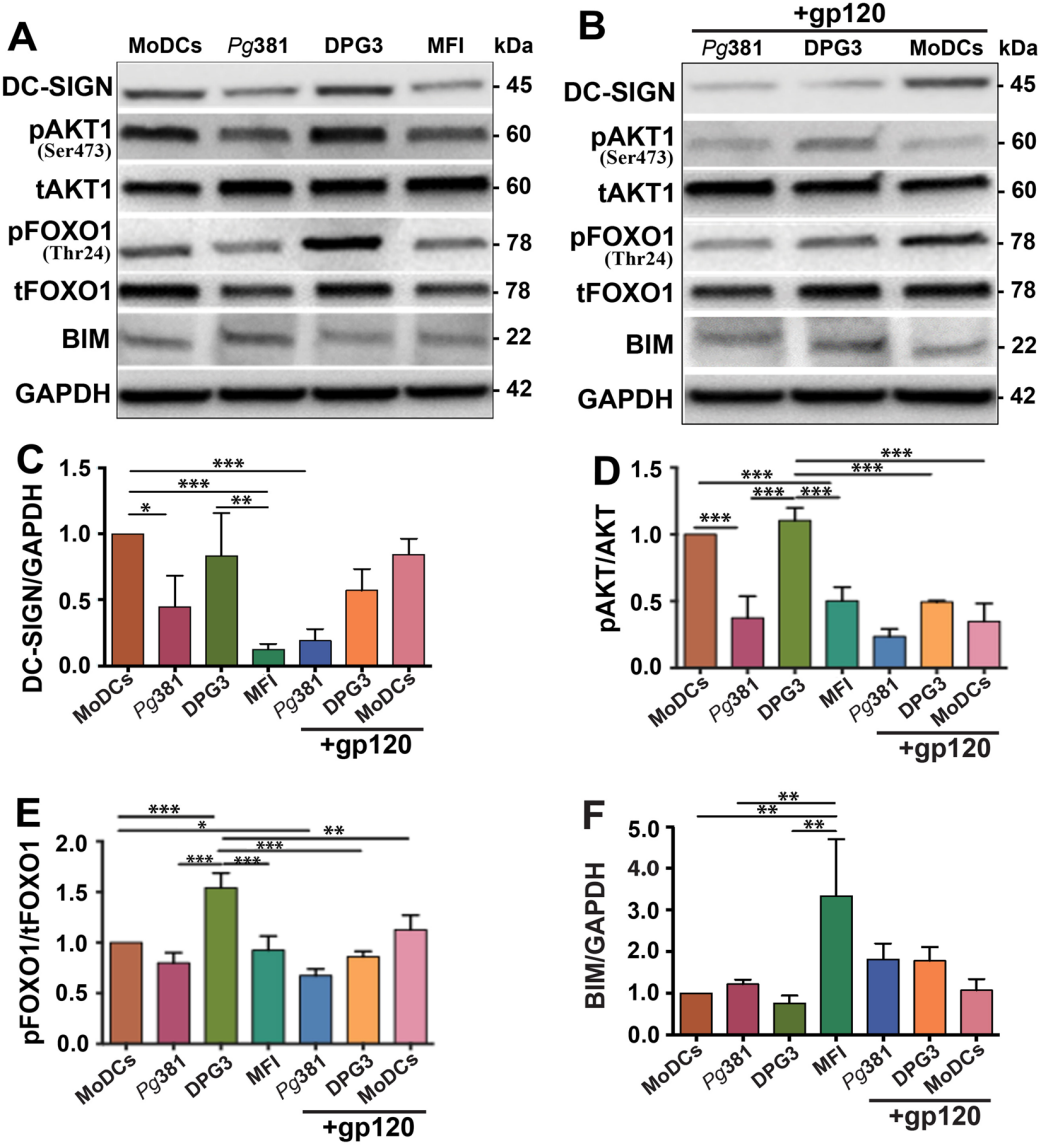
DPG3 activated DC-SIGN, AKT, regulated FOXO1 phosphorylation/expression was required for the survival effect of DPG3 in MDDSCs. Immature monocytes isolated from human peripheral blood mononuclear cells (PBMCs) were infected with *Pg*381, DPG3 and MFI for 6 hours at 1 MOI. (**A**) Immunoblot analysis of DC-SIGN, p/tAKT, p/tFOXO1 and BIM in MDDSCs and MoDCs with GAPDH as loading control. Immunoblot detected increased expression of DC-SIGN, p/tAKT, p/tFOXO1 and decreased expression of BIM in DPG3- infected MDDSCs compared with uninfected control MoDCs. (**B**) DC-SIGN was blocked with HIV-gp120 (6ug/ml) for 30 minutes before infection. Notably, DC-SIGN expression and internalization was abolished by its antagonist HIV-gp120 and consequently the downstream signaling cascade also blocked as evident by dephosphorylated or decreased expression of pAKT (**B**,**D**), pFOXO1 (**B**,**E**) and increased expression of BIM (**B**,**F**). (**C**–**F**) Quantification analysis of DC-SIGN (**C**), and relative ratio of p/tAKT (**D**), p/tFOXO1 (**E**) and BIM (**F**) after normalized with the internal control GAPDH. Results are from one representative of three independent experiments. Data were analyzed by performing one-way ANOVA with Tukey’s post hoc using Prism GraphPad. *P ≤ 0.05, **P ≤ 0.01, ***P ≤ 0.001.

We next examined by immunoblot, protein levels of pAKT and immaturity DC marker DC-SIGN in MDDSCs induced by DPG3 relative to *Pg*381 and MFI (totally lacks Mfa1) or uninfected MoDCs (Fig. 1A,B). DPG3 induces more phosphorylation of AKT1, relative to FimA expressing strains *Pg*381 and MFI (Fig. 1A,D). We posited that this could be promoting, not only survival of DPG3 through evasion of autophagy via DC-SIGN routing^44^, but also anti-apoptotic signaling. Since BIM, a pro-apoptotic gene controlled by FOXO1, promotes DC apoptosis^37^, we assessed relative p/tFOXO1 induction by DPG3 and *Pg*381 in MDDSCs by immunoblot (Fig. 1A and E). Interestingly, the most anti-apoptotic inducing strain DPG3, considerably induced pFOXO1 whereas BIM protein expression decreased compared with control MoDCs (Figs 1A,F and S2C). Then, to address the role of DC-SIGN in activation of this signaling pathway, HIV-gp120 was used to block DC-SIGN as we have reported^34,46^. By immunoblot we detected increased DC-SIGN receptor expression by DCs induced by DPG3 relative to *Pg*381 and MFI or uninfected control (Fig. 1A,C), whereas blocking DC-SIGN with HIV-gp120 prior to infection reduced DPG3 (Mfa1) mediated-induction (Fig. 1B,C). However, it is noteworthy that DC-SIGN expression was not altered by gp120 alone in control MoDCs (Fig. 1B) and was thus a consequence of blocking *P. gingivalis* entry and/or activation of the DC-SIGN signalosome^34,46^. We should emphasize that DPG3 stimulation led to AKT serine473 (Ser473) phosphorylation which regulated FOXO1 threonine24 (Thr24) phosphorylation and expression in the DCs (Fig. 1A). Activation of AKT activity by Ser473 phosphorylation of its expression promotes cell survival through FOXO1 Thr24 phosphorylation. To verify the role of AKT in this pathway, DCs were co-treated with gp120, which impaired DC-SIGN–mediated survival signaling (Fig. 1B,D) and pFOXO1 (Fig. 1B,E). We also found that gp120 abolished this pathway in *P. gingivalis381*-induced MDDSCs by significantly decreased expression of DC-SIGN, pAKT1 and pFOXO1 functional proteins (Fig. 1C–E), whereas the apoptotic protein BIM was increased (Fig. 1B,F). These data thus indicate a role of DC-SIGN in the DPG3–induced pAKT1-pFOXO1 mediated apoptosis resistance and immunosuppressive signaling pathway in MDDSCs.

### Short-term murine oral infection with *P. gingivalis* fimbriae mutants activate immunosuppressive genes in blood and splenocytes

**Figure 2:**
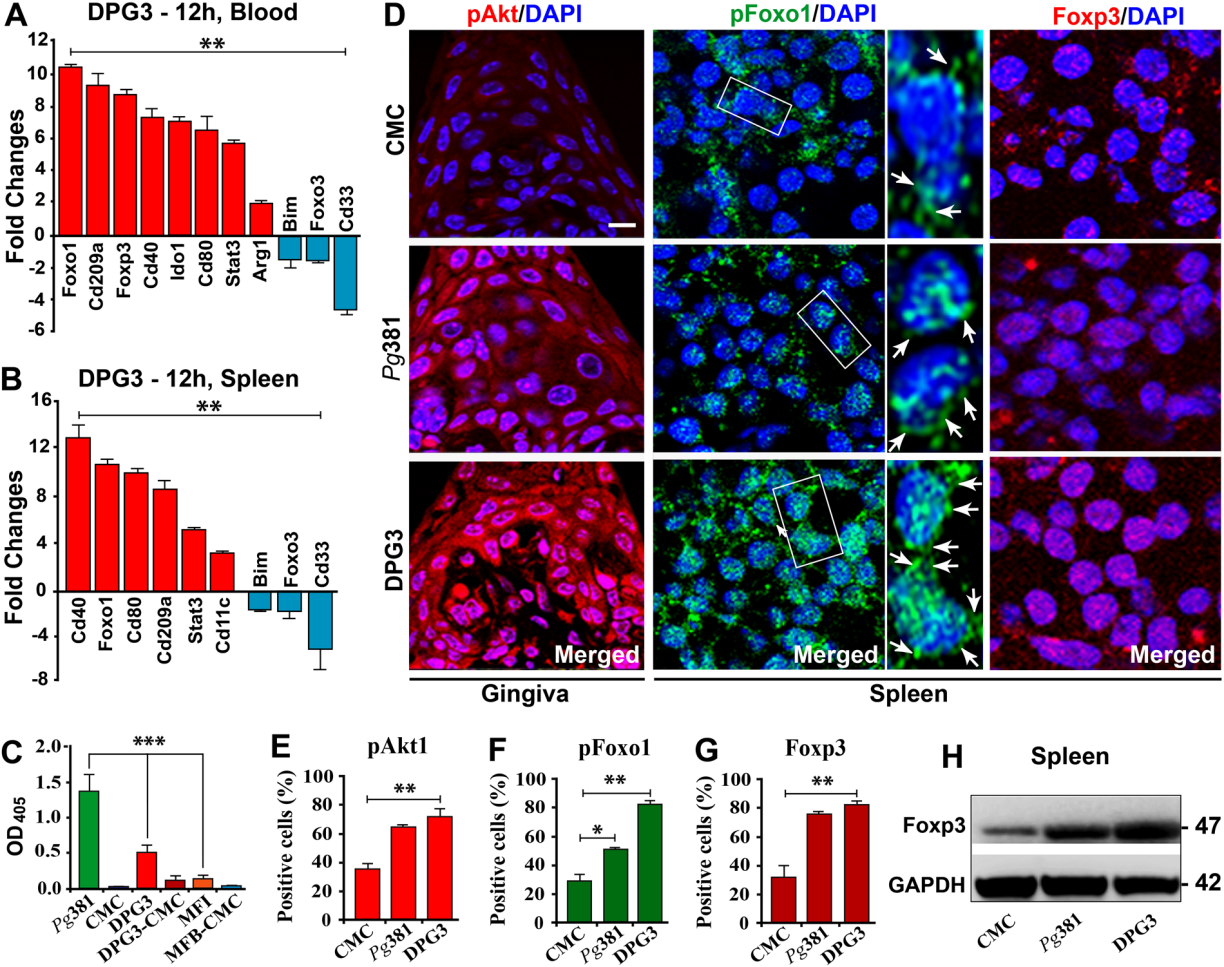
Oral infection of mice with *P. gingivalis* induces pAkt/pFoxo1/Foxp3 mediated immunosuppression. The gene expression profile shows differential response of blood (**A**) and splenocytes (**B**) isolated from DPG3-infected mice (n = 3) at 12 hours compared with negative control group (n = 3; 2% CMC without bacteria). Total of 36 mice were sacrificed at 1, 12 and 24 hours (from groups:- 1. CMC; 2. *Pg*381; 3. DPG3 and 4. MFI), and blood (refer Fig. S3) and splenocytes were collected and genes quantified by TaqMan® array. (**C**) *P. gingivalis* DPG3-fimbriae mutants induce lower IgG antibody titer after 4-weeks oral infection. Sera from infected mice (n = 12) was collected after 4 weeks of infection with *Pg*381, DPG3, MFI and CMC control to determine total IgG titers against respective formalin-killed *P. gingivalis* strains, measured by ELISA. DPG3 was shown to induce reduced antibody titer compared to *Pg*381, whereas MFI induced minimal IgG. ***P < 0.001, ANOVA. (**D**) Immunofluorescence analysis (IFA) of pAkt1 protein expression in gingival interproximal papilla area between M1&M2, pFoxo1 and Foxp3 in spleen tissue from DPG3, *Pg*381 infected mice, compared with uninfected CMC group (refer Fig. S4). Arrow shows Foxo1 phosphorylation and nuclear exclusion of pFoxo1 (middle panel). For independent channels refer Fig. S4. Scale bar: D 20 µm. Images are representative of three independent experiments. (**E**–**G**) Quantification analysis of pAkt, pFoxo1 and Foxp3 expression in DPG3 and *Pg*381 compared to CMC infected mice gingiva (**E**) and splenocytes (**F** and **G**). (**H**) Immunoblot shows increased expression of Foxp3 in *Pg*381 and DPG3 infected mice splenocytes, compared with CMC. *P ≤ 0.05, **P ≤ 0.01.

We next tested the ability of oral infection with *P. gingivalis* to induce this immunosuppressive pathway in gingival tissue, blood and secondary lymphoid organ, spleen of mice. Gene expression profiles of isolated blood (Fig. 2A) and splenocytes (Fig. 2B) of BL6 mice orally infected with *P. gingivalis* or its fimbriae deficient strains show distinct *in-vivo* responses depending on fimbria expression (Fig. S3). The strongest immunosuppressive responses were induced by Mfa1 + strain DPG3 after 12 hours of oral infection, including upregulation of Foxo1, Cire/Cd-209a, Cd40, Cd80 and Stat3 in blood (Figs 2A and S3B) and splenocytes (Fig. 2B), whereas Ido1 and Foxp3 were only induced in blood, but Bim, Foxo3 and Cd33 were downregulated in both. Serum IgG responses to *P. gingivalis*381 were also dampened by oral infection with DPG3 (Fig. 2C). These results emphasize the key role of *P. gingivalis* and its Mfa1 fimbriae type in early immunosuppressive responses.

### Oncogene Akt1 induction and immunosuppressive state in DPG3-infected mice

We further hypothesized that oncogene Akt1^47^ was activated locally in mice orally infected with Mfa1 + *P. gingivalis*. Immunostaining (Figs 2D-left panel, E and S4A) shows pAkt1 localized mostly at the level of sulcular epithelium and subjacent connective tissue, consistent with gingival inflammation, which may lead to angiogenesis and oncogenesis. Also, increased pFoxo1 (Fig. 2D-middle panel: phosphorylation and nuclear exclusion of pFoxo1 protein indicated by arrow marks, 2F and S4B-left panel) expression was noted in DPG3 and *Pg*381 infected mouse splenocytes, compared to CMC treated. It is interesting to note that Foxp3 an important immunosuppressive factor in regulatory T cells (T_regs_), is significantly increased in the gingiva of *P. gingivalis* and DPG3 infected mice (Fig. 2D,G and S4B-right panel), and in spleen of DPG3 and *Pg*381infected mice by immunoblot compared to uninfected CMC control. These data suggest that the fimbriae expression pattern of *P. gingivalis* strain type may play a key role in oncogenic progression and immunosuppression.

### Distinct and direct induction of proliferation of oral cancer cell lines by *P. gingivalis*

**Figure 3:**
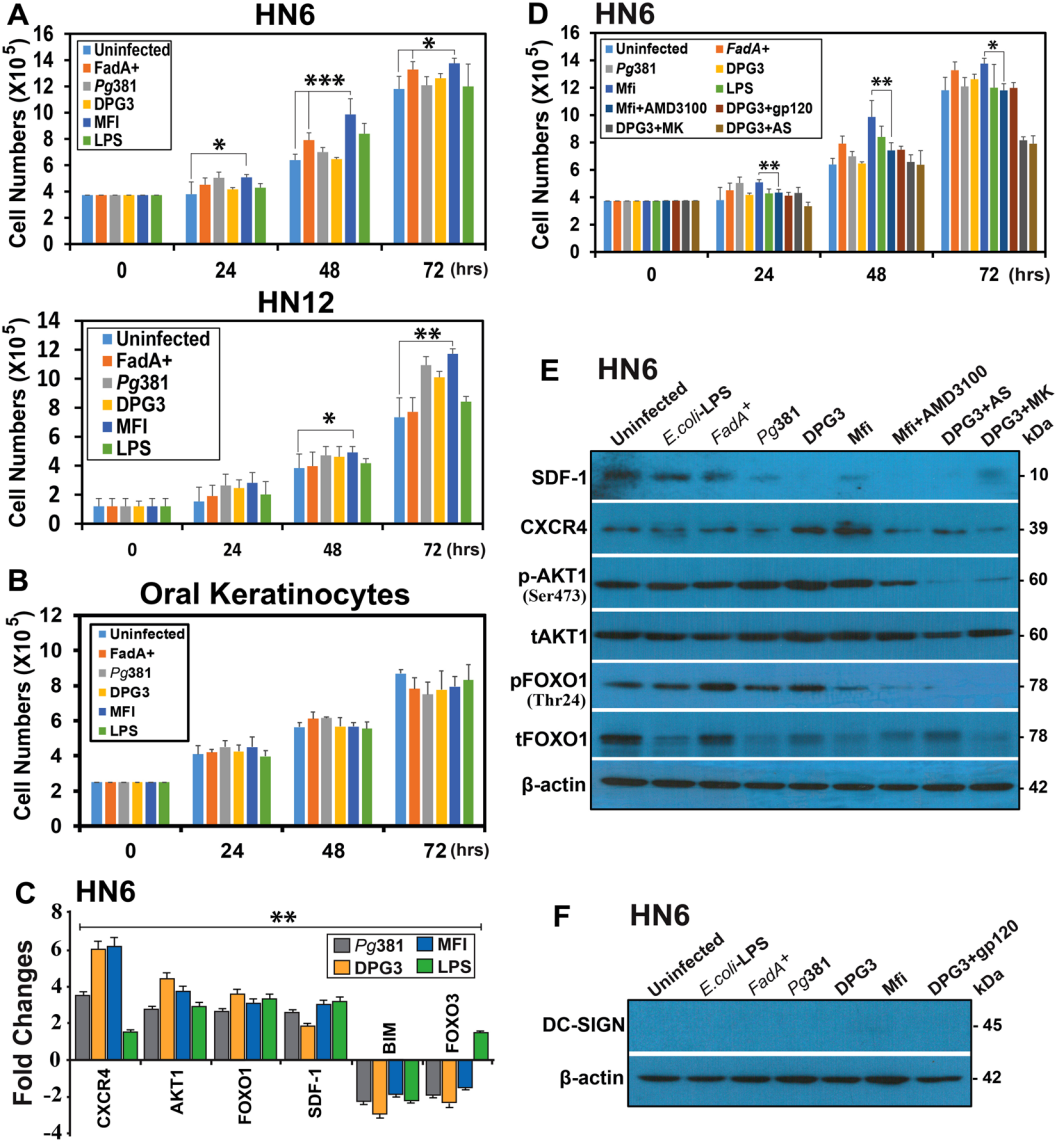
FimA+Mfa1^−^ *Pg* strain MFI stimulates proliferation of Human oral squamous carcinoma cells via CXCR4. (**A**) Pg-MFI (Mfa1^−^/FimA^+^) stimulates proliferation of human head and neck OSC cell lines HN6 and HN12, compared to uninfected cells or those incubated with *E. coli*-LPS, but not the noncancerous (**B**) oral keratinocytes (hTERT HAK Clone 41). For other noncancerous ARPE and MEF cells refer Fig. S5. (**C**) Wild-type *Pg381*, DPG3 and MFI induces expression of CXCR4, AKT1, FOXO1 and SDF1 in HN6 cells at 24 hours, whereas decreases the expression of BIM and FOXO3, as determined by qPCR. (**D**) Suppression of MFI-stimulated cell growth by inhibiting CXCR4. MFI-stimulated HN6 growth was inhibited by the CXCR4-antagonist AMD3100, but weakly or not by the DC-SIGN inhibitor gp120 after infected with DPG3. (**E**) MFI activates CXCR4-mediated Cellular Signaling in the direction of oncogenesis. Immunoblot analysis was used to detect the following proteins SDF-1/CXCR4, p/tAKT1, and p/tFOXO1 in *E. coli*-LPS, FadA^+^, *Pg*381, DPG3 and MIF treated HN6 cells compared with uninfected control, but pAKT1, pFOXO1 and SDF-1/CXCR4, were inhibited by their respective inhibitors such as MK-2206 (MK), AS1842856 (AS) and AMD3100. (**F**) DC-SIGN was not detectable by immunoblot in HN6 cells, thus confirming role of CXCR4 in FimA^+^ MFI stimulation of proliferation. The results are presented (as the mean ± SD) from one representative of four independent experiments. *P ≤ 0.05, **P ≤ 0.01, ***P ≤ 0.001.

We further hypothesized that activation of this pAKT-pFOXO1 signaling pathway by *P. gingivalis* would promote direct proliferation of OSCCs *in-vitro*. The results show that human head and neck oral squamous carcinoma cell (OSCCs) lines HN6 and HN12 were stimulated to proliferate, but not by Mfa1 + DPG3, as we suspected, but by FimA^+^MFI, relative to uninfected control or *E-coli-*LPS stimulated, at 24, 48 and 72 hours (Fig. 3A). No proliferation was induced in the noncancerous human oral keratinocytes (hTERT HAK Clone 41) (Fig. 3B), human retinal pigment epithelial (ARPE) or mouse embryonic fibroblasts (MEF) cells (Fig. S5). Apoptotic cells were observed in *Pg* strain- infected ARPE cells at 48 and 72 hours, compared with uninfected control (Fig. S5). Stimulation of proliferation by FimA^+^MFI was superior to *Fad*A^+^ oncogenic *Fusobacterium nucleatum*^48^ at 48 and 72 hours. Thus FimA^+^MFI strain induced the most direct effect on proliferation of cancer epithelial cell lines. In contrast, noncancerous oral keratinocytes, ARPE and MEF cells were not stimulated by *P. gingivalis*381. Real-time quantitative PCR (qPCR) analysis at 24 hours shows > 2-fold induction of angiogenesis, oncogenic and proliferation related genes (CXCR4, SDF1, AKT1, FOXO1) in HN6 cells, whereas apoptosis (BIM) and tumor suppressor (FOXO3) related genes were down-regulated by all strains (Fig. 3C).

### FimA-expressing *P. gingivalis* promotes OSCC proliferation via CXCR4

As FimA targets CXCR4 on macrophages^49^, and is a co-receptor for the entry of specific strains of human immunodeficiency virus type I, we treated HN6 cells with CXCR4 inhibitor AMD3100, prior to co-culture with FimA^+^MFI. The results demonstrate a marked inhibition of HN6 proliferation (Fig. 3D) and comparable with control. In contrast blocking DC-SIGN mediated uptake of DPG3 using HIV-gp120 did not ablate proliferation (Fig. 3D). These results elucidate the critical role of CXCR4 and thus possibly FimA, in promoting OSCC cell growth.

### FimA activates CXCR4-mediated cellular signaling in OSCCs

Next, we tested whether the HN6 cells express DC-SIGN, since blocking DC-SIGN mediated uptake of DPG3 by gp120 was not able to block the proliferation of OSCCs. Immunoblot analysis confirmed that HN6 cells constitutively express CXCR4 (Fig. 3E), but not DC-SIGN (Fig. 3F); moreover, CXCR4, but not DC-SIGN is upregulated by *P. gingivalis*381 (Fig. 3F). Thus, differential receptor expression helps to explain the distinct roles of Mfa1 and FimA fimbriae in MDDSCs versus epithelial cells. Nonetheless, CXCR4 upregulation was also accompanied by increased phosphorylation of AKT1 (Ser473), pAKT1 accumulation in the cytoplasm, and translocation into the nucleus, resulting in activation of pAKT1-regulated transcription, as evidenced by increased expression and its Thr24 phosphorylation of transcription factors FOXO1 (Fig. 3E). The CXCR4 inhibitor AMD3100 reduced expression of oncogenic proteins SDF-1/CXCR4 expression, as well as pAKT1 and pFOXO1 activation (Fig. 3E), but it is interesting to note that the CXCR4 expression is comparable with the uninfected control. Moreover, control inhibitors of pAKT1 and pFOXO1, MK-2206 2HCL (MK) and AS184285 (AS), respectively, blocked activation of respective mediators (Fig. 3E). It is noteworthy that DPG3 also induces expression of CXCR4 in HN6 (Fig. 3E), however, the DC-SIGN expression was not detectable by immunoblot after exposure to *Pg*381and their mutant strains (Fig. 3F), thus, this data suggests that in addition to the receptor function of CXCR4 protein, its role as a metastasis mediator is only activated upon ligation by the cognate ligand, in this case, FimA. Although CXCR4 expression is higher in both DPG3 and MFI stimulated HN6 cells, DPG3 lacks FimA for ligation of CXCR4 leading to proliferation of OSCCs. Over all, these results suggest that CXCR4, provided it is ligated by FimA, is required for activation of the same pathway in OSCCs as in MDDSCs, namely, pAKT1-pFOXO1 pathway involved in immunosuppression and oncogenesis.

### Systemic immunosuppressive and oncogenic state in CP patients

**Figure 4:**
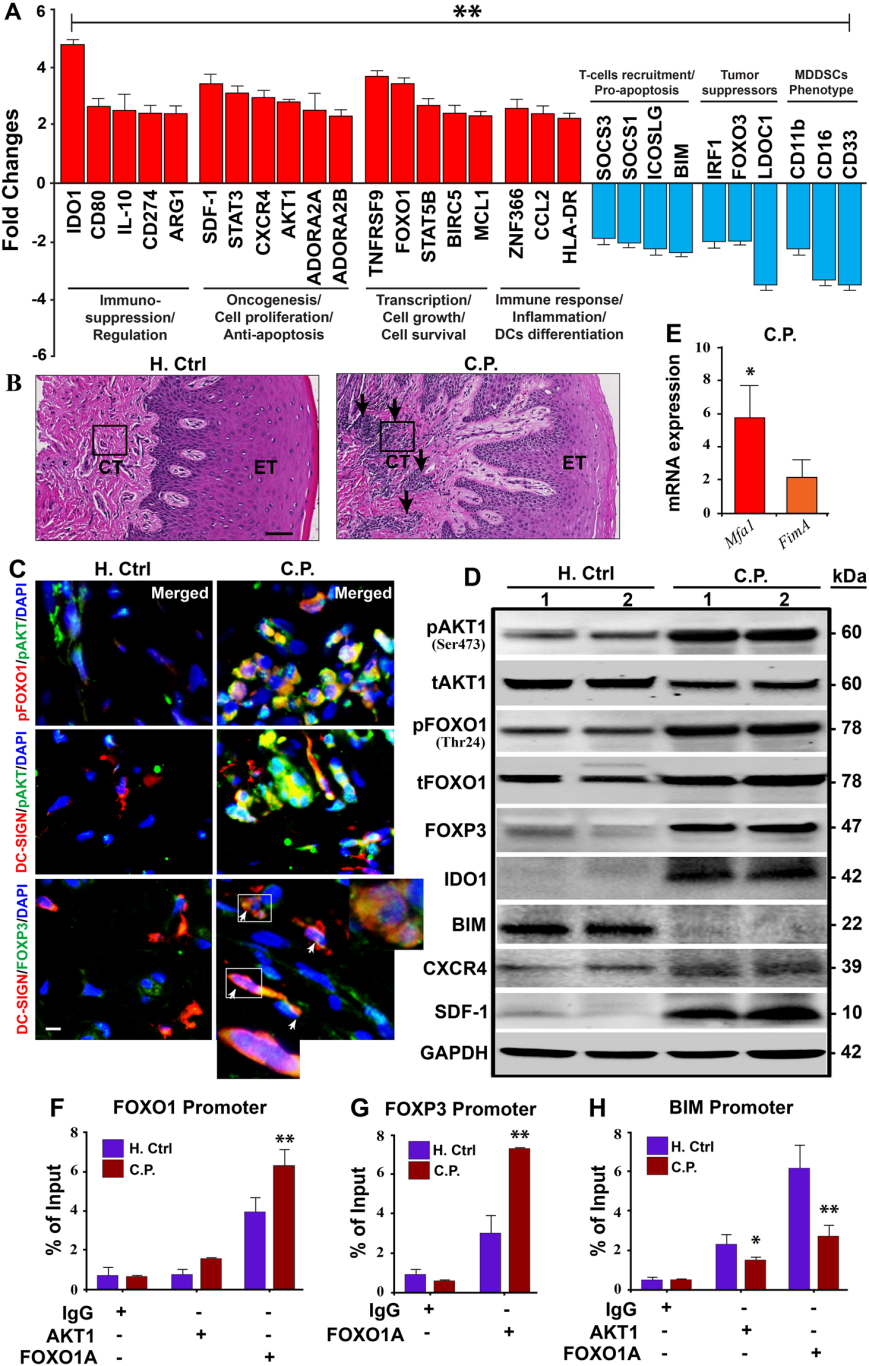
Enriched blood panDCs and gingival tissues of CP patients reveal immunosuppressive state. (**A**) 29 genes associated with transcriptional regulation, inflammation, immunosuppression, cell proliferation, anti-apoptosis and other homeostatic functions were identified from the RNAseq data. Bar graphs show both up- (red) and down- (blue) regulation of genes (≥±2 fold) in *ex-vivo* isolated and panDC-enriched blood samples from CP patients compared to healthy controls confirmed by TaqManPCR. The overall validation data shows the same trend as in RNAseq analysis. (**B**) H&E of healthy control and CP patient’s gingival tissue. The arrows indicate the infiltrating cells of inflammatory responses; (20X), CT- Connective Tissue, ET- Epithelial Tissue. Results are representative of two experiments (n = 9/group). (**C**) Co-localized expression of pAKT1/pFOXO1, pAKT1/DC-SIGN (receptor for Mfa1) and DC-SIGN/FOXP3 (arrowhead) in gingival connective tissue (marked in b) from CP, compared with healthy control. Images are representative of three independent experiments (Scale bar- 100 µm; pls refer Fig. S6 for independent channel and their quantification measurement of co-localization. (**D**) Immunoblot analysis of p/tAKT1, p/tFOXO1, FOXP3, IDO1, BIM, CXCR4 and SDF1 in gingival tissue of CP compared with healthy control. (**E**) *Mfa1* and *FimA* mRNA in *ex-vivo* isolated panDCs from CP patients and Healthy control and normalized with the internal control. (**F–H**) Regulation of FOXO1, FOXP3, BIM transcription by FOXO1A or pAKT1. Immunoprecipitation of chromatin from the human FOXO1 (**F**), FOXP3 (**G**) and BIM (**H**) locus in mixed immune cell population from gingival tissues of CP and healthy control with anti-pAKT1 or anti-FOXO1A, followed by qPCR analysis of immunoprecipitants; results are presented relative to input by immunoprecipitation with isotype-matched control antibody. Data are representative of six independent experiments. *P ≤ 0.05, **P ≤ 0.01.

To determine if this immunosuppressive and oncogenic pathway is active in humans with chronic periodontitis (CP), blood and gingival biopsy samples were obtained from CP and healthy control subjects. RNA of enriched DCs was analyzed for differentially expressed genes in CP patients normalized to healthy controls. The genes were cross-referenced with gene database from RNAseq of *in-vitro* MoDC infection model^41^. Of major significance were genes specifically induced in MoDCs *in-vitro* by DPG3, namely, CD274, IDO1, FOXO1, AKT1, CD209 (DC-SIGN), CXCR4, IL-10, FOXP3, CCL2 (MCP-1), STAT3 and STAT5B. Many of these same genes, particularly involved in immunosuppression (IDO1, IL-10, CD274, ARG1 and CD80), oncogenesis (CXCR4, SDF-1, AKT1 and STAT3) were highly induced in *ex-vivo* isolated blood panDCs of CP patients (Fig. 4A), whereas SOCS3 and SOCS1 were downregulated, which could render DCs more tolerogenic. Of vital note is the gene for IDO1, which was upregulated 5-fold relative to controls, consistent with our previous *in-vitro P. gingivalis* infection model^43^. Histopathological analysis of gingival tissues from representative CP subject, compared with healthy control, shows chronic inflammatory infiltrates consisting of lymphoid and myeloid cells in the CP connective tissues (Fig. 4B). Preferential induction of dysbiotic transcription factor pAKT1 in CP gingival tissues by myeloid dendritic cell subpopulation, as confirmed by immunofluorescence analysis (Figs 4C and S6) showing pAKT co-localization with pFOXO1 and DC-SIGN (DC’s marker) as well as the immunosuppressive T_reg_ protein FOXP3 co-localized with DC-SIGN, compared with healthy control, supporting involvement of pAKT1 in dysbiosis. Interestingly, immunoblot shows increased pAKT1, pFOXO1, FOXP3, IDO1 and decreased BIM proteins (involved in immunosuppressive and oncogenic pathway) in CP gingival tissues compared with healthy controls (Fig. 4D). These data support an immunosuppressive state in severe CP patients prone to the oncogenic process.

### *P. gingivalis* and its Mfa1 fimbrial genotype increased in enriched blood DCs from CP patients

To identify the presence of *P. gingivalis* and its relative Mfa1 + fimbrial expression in CP, we analyzed mRNA from enriched blood panDCs of CP patients and healthy controls. The healthy control DCs contained no detectable *P. gingivalis* 16 s rDNA, so we focused on CP samples. The results (Fig. 4E) show higher mRNA-expression of *Mfa1*, relative to the other fimbriae type, *FimA*, inside blood DCs. When considered in the context of immune homeostatic functions disrupted by DPG3 *in-vitro* (Fig. 1), *in-vivo* (Fig. 2) and in CP samples (Fig. 4), this supports Mfa1 as an important dysbiotic factor that promotes a systemic immunosuppressive and anti-apoptotic state. This result also suggests that *Pg*381 may adapt physiologically and genetically to the *in vivo* environment of CP by upregulating immunosuppressive genes.

### Role of FOXO1, FOXP3 and BIM target genes in FOXO1 and AKT1 signaling in CP patients

To confirm the mechanistic role of AKT1 or FOXO1 in regulation of FOXO1, FOXP3 and BIM transcription in CP tissues *in situ*, we searched for evolutionarily conserved FOXO-binding sites in human FOXO1, FOXP3 and BIM loci. Using the rVISTA tool, we found putative FOXO1 and FOXP3 -binding sites in previously defined conserved noncoding sequences of FOXO1, FOXP3, and BIM promoter region (Table S4). We reiterate the observation of dramatically higher levels of pFOXO1 in DPG3 infection induced MDDSCs compared to control monocytes (Fig. 1) suggesting that FOXO1 expression is negatively regulated by PI3K–AKT signaling pathway. To further corroborate FOXO1 as a target for AKT1 in cells from gingival tissues of CP, we examined FOXO1 as well as FOXP3 and BIM expression and their nuclear localization using antibodies to FOXO1 (anti-FOXO1) or anti-AKT1. Chromatin Immunoprecipitation (ChIP) assays demonstrated genomic fragments containing the proximal FOXO1 (Fig. 4F) promoter regions in the DNA upstream of the translation start site selectively enriched with the pAKT1 antibody, whereas the BIM (Fig. 4H) was significantly decreased in gingival tissues. We next tested if FOXO1 regulates the expression of FOXP3 and BIM in cells from gingival tissues, selectively enriched and repressed for genomic fragments containing the region in FOXP3 (Fig. 4G) and BIM (Fig. 4H) promoters, respectively. This finding describes a critical role for an evolutionarily conserved AKT1 and FOXO1 binding elements in control of FOXO1, FOXP3 and BIM promoter activity and supports their role as target genes for immunosuppression and dysbiosis in CP.

### Oncogenic profile confirmed in oral tissues from *P. gingivalis* infected CP patients

Since MDDSCs provide anti-apoptotic, immunosuppressive functions *in-vitro* and *in-vivo*; we further analyzed the genes and proteins related to oral cancer in *P. gingivalis* infected CP patient tissues by qPCR and immunoblot, respectively. The results delineate increased expression of angiogenic and oncogenic markers CXCR4, SDF-1, and decreased expression of tumor suppressor genes, IRF1, LDOC1, and FOXO3 in CP patient tissues (Fig. 4A). A surprise consequence was the similar responses observed in CP patient samples as MDDSC phenotypic markers (CD16^−low^, CD11b^−low^ and CD33^−low^) (Fig. 4A) was also consistent with the results obtained *in-vitro* (Fig. S1A,B). Interestingly, immunoblot analysis shows increased expression of oncogenic markers CXCR4 and SDF-1 in oral tissues of CP compared with healthy controls (Fig. 4D), thus these data demonstrate that the pathobiont *P. gingivalis* activates anti-apoptotic pathway promoting immune suppression and possibly, oncogenesis.

## Discussion

We report here two distinct, though linked mechanisms activated by the oral pathobiont *P. gingivalis* that may possibly promote oral and other cancers: (a) immunosuppression through induction of MDDSCs, predominantly dependent on the DC-SIGN targeting Mfa1 fimbriae and (b) oncogenic response, predominantly through the CXCR4-targeting FimA fimbriae. Several recent reviews discuss the various cancers associated with CP, including gastrointestinal, pancreatobiliary tract^50^ and oral squamous cell carcinoma (OSCC)^4,51,52^. OSCC represents the sixth most common malignant tumor. Nearly, 90% of cancers of the upper aerodigestive tract are SCCs^53,54^. The role of oral microbiome, including *P. gingivalis*, *Fusobacterium nucleatum (Fn)*, *Streptococcus gordonii* (*Sg*) and other species in oral, esophageal, pancreatic, gastrointestinal and colorectal cancers has also been described^46,48,54–56^. *P. gingivalis* promotes a mucosal dysbiosis, disrupting immune homeostasis through unclear mechanisms. *P. gingivalis* colonizes pre-malignant lesions of the esophagus^57^ and OSCC^54^ and has been called a keystone pathogen due to its outsized influence on the oral commensal microflora^55^. An invasive pathogen, *P. gingivalis* infects myeloid DCs^46^, endothelial cells^58^ and epithelial cells^59^ in periodontitis. *P. gingivalis* is able to survive in the host environment by evading autophagy in DCs^44^ and is disseminated within migratory blood DCs^15^. The DCs thus subjugated are resistant to apoptosis and are re-directed to the periphery^45^. The present study shows how immune homeostasis by this periodontal infection is disrupted, contributing to dysbiotic diseases of the upper^11^ and lower^60^ GI tract.

MDDSCs induced by Mfa1 + *P. gingivalis* share monocytic lineage with MDSCs and with conventional DCs. Common function-related markers in MDDSCs and MDSCs include IL-10, TGFβ and PD-L1^61^, as well as STAT3, CD15 and ARG1 in human MDSCs^22^. A recent review highlights the importance of phenotypic and transcriptional-profiling to redefine the DC lineage^62^. As blood panDCs of CP patients contained *P. gingivalis* Mfa-1, transcriptional profiling of panDCs was performed and cross-referenced with transcripts induced by infection of human monocytes and mice with Mfa1 + *P. gingivalis*. Most notable were of genes involved in DC differentiation, immune regulation and regulation of apoptosis, markedly FOXO1. Examination of gingival tissues of CP patients by functional genomics ChIP assay specifically links FOXO1 proteins with the DNA element of FOXP3, indicating that FOXP3 is a direct FOXO1 target gene in CP tissues. This is consistent with the demonstrated ability of MDDSCs to induce T_reg_. The progression of CP in humans is related to over-expression of FOXP3 and IL-17^63^ and with uptake of *P. gingivalis* by blood myeloid-derived DCs *in-vivo*^15^. In addition, the transcription factor STAT5B, also activated by Mfa1 + *P. gingivalis* strain DPG3, binds to regulatory elements in the FOXP3 locus and may regulate FOXP3 expression. Thus, FOXO1 proteins appear to be primary regulators of MDDSC plasticity and T_reg_ cell lineage and are induced by *P. gingivalis* infection. The production of IDO1 in chronically inflamed sites, as in CP^64^, can block Th1 and Th17 effector differentiation, T cell apoptosis and promote T_regs_. Immature or regulatory DCs can produce IDO^65^ capable of biasing the immune system towards tumor growth support. High rates of necrotic and apoptotic cell death have been observed in IDO-KO mice after *P. gingivalis* LPS challenge compared to WT mice^26^. We showed that IDO was involved in both anti-apoptotic and immune suppressive response of MDDSCs. In addition, we have also shown increased expression of immunosuppressive cytokines IL-10, TGFβ^66^, immunosuppressive molecule PD-L1^67,68^ and Survivin (BIRC5)^69^, which influence tumor progression in many types of cancers. Strikingly, we found that in CP patients, there was increased expression of immunosuppressive, angiogenic and oncogenic markers, and decreased expression of tumor suppressors in the gingiva at mRNA and protein level. Another interesting finding of this study was increased expression of *Mfa1* at mRNA level in CP patient blood panDCs, which might be one of the main roles of *Pg*381 dissemination, wherein the bacterium adapts physiologically or genetically to their local environment.

Our results show a key role for DC-SIGN in *P. gingivalis* Mfa1-mediated activation of STAT3, which phosphorylates AKT1 (pAKT1) for full activation. Phospho-AKT1 suppresses BIM, promoting apoptosis resistance and monocyte differentiation into MDDSCs. The expression of active forms of FOXO family members in dividing cells promote cell cycle arrest at the G1/S boundary, which is a significant cellular mechanism by which FOXO promotes tumor suppression^70,71^. Phospho-AKT1 in turn phosphorylates and inactivates FOXO1 which further suppress BIM, a key FOXO target gene that mediates the ability of FOXO to induce cell death^72^. It is interesting to note that AKT-FOXO1 regulates the BIM and transcriptionally inactivates FOXO1 (pFOXO1) leaving no opportunity to bind with BIM to subsequently mediate apoptosis upon the persistent stimulation of *Pg* strains. Phosphorylated FOXO1 also regulates FOXP3 expression through its feedback regulatory loop mechanism as per the consistent and continuous stimulation of *Pg* strains in MDDSCs, to promote apoptosis resistance and immunosuppression. This pathway (DC-SIGN-pAKT1-pFOXO1-BIRC5-STAT3-IDO1-FOXP3) culminates in MDDSC differentiation, T_regs_ activation and IDO-mediated immunosuppression, which may regulate cellular processes leading to immune escape.

We have also elucidated here an oncogenic mechanism whereby *P. gingivalis* activates OSCCs and have identified the molecular target. We have shown increased expression of metastasis markers CXCR4, SDF-1^73,74^ and decreased tumor suppressor molecules LDOC1 and FOXO3, induction of oncogenes and promotion of tumor growth. Immunoblot analysis further confirmed increased expression of CXCR4 and SDF-1 in oral tissues of CP compared with healthy controls. Further, strain MFI (FimA^+^Mfa1^−^*Pgingivalis*) stimulates significant proliferation of CXCR4-expressing OSC (HN6, HN12) cells, but not those of noncancerous cells (oral keratinocytes, ARPE and MEF), which indicate that the interactions between FimA and CXCR4 play a primary role in promoting OSCCs tumor growth through the pAKT1-pFOXO1 dependent pathway(s). In addition, the available evidence indicates that the broad somatic deletion of FOXO1/3/4 in mice results in thymic lymphomas and hemangiomas^75^, specifying that the FOXO family functions as a tumor suppressor in mice^70^. Interestingly, we found increased expression of pFOXO1 (transcriptionally inactive) and decreased expression of FOXO3 in *in-vitro*, *in-vivo* and *ex-vivo* samples, thus supporting the findings that FOXO family members can promote tumor progression.

**Figure 5:**
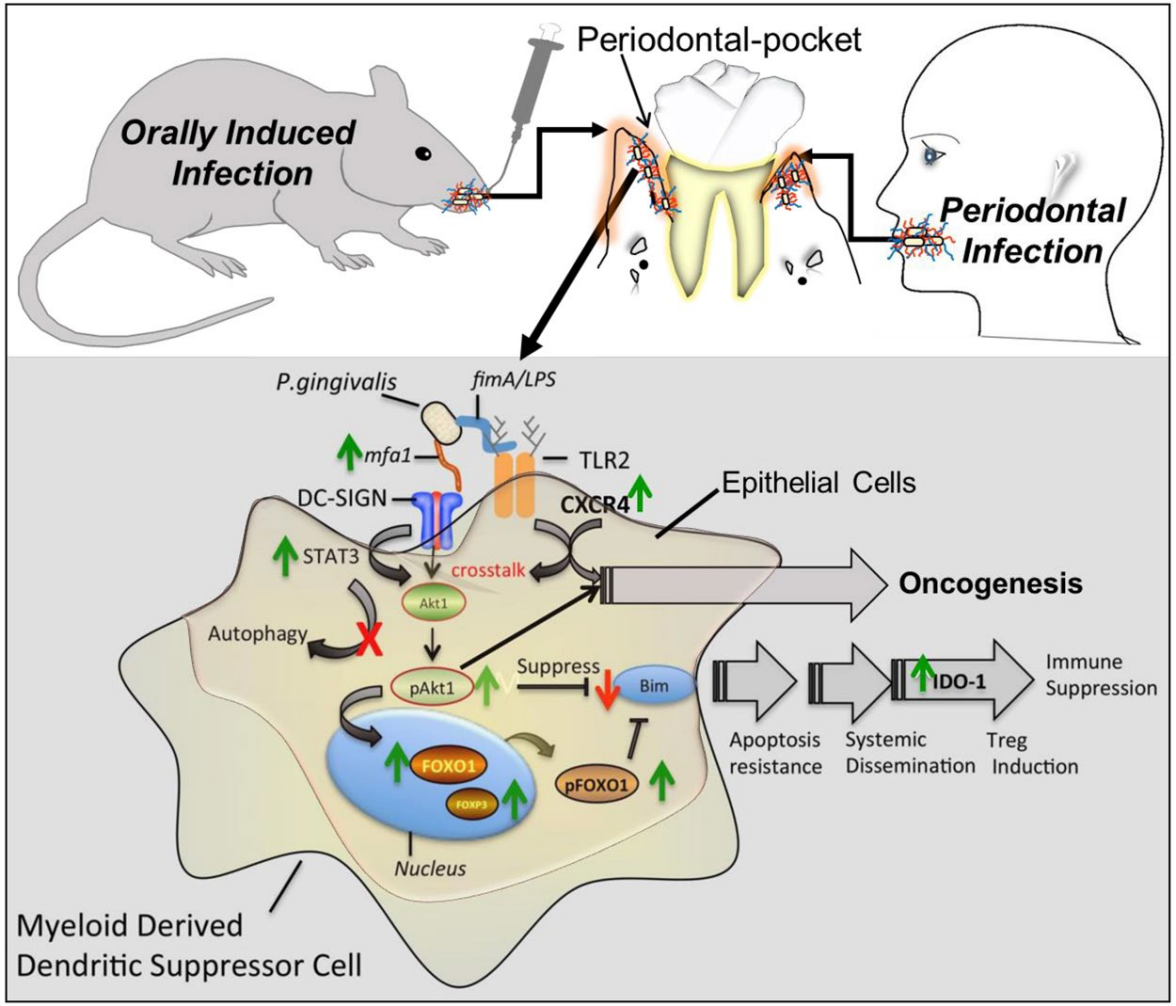
Model of immune homeostasis disruption and dysbiosis by *P. gingivalis*. DC-SIGN - TLR2/CXCR4 crosstalk activated by ligation via *P. gingivalis* Mfa1 - FimA, respectively, activates STAT3, resulting in downstream AKT1 activation that phosphorylates and inactivates FOXO1. Direct suppression of apoptotic activity through BIM also occurs. DC-SIGN routes *P. gingivalis* into non-autophagosomal compartments where they survive. Upon phosphorylation by AKT1 the FOXO proteins are excluded from the nucleus and are sequestered or degraded. FOXO inactivation contributes to extended cell survival as FOXO members activate genes encoding pro-apoptotic molecules including Bcl-2-member BIM. The resultant MDDSCs are highly resistant to apoptosis and IDO-1 competent, traveling to local and distant sites bearing the bacteria and promoting immunosuppression through induced-T_regs_ (FOXP3). In addition to the immunosuppressive route through DC-SIGN by DPG3 (Mfa1), \FimA (MFI) leads the direct oncogenesis route through CXCR4.

In conclusion, our study supports a novel apoptosis resistant pathway (Fig. 5) that promotes both immunosuppression and oncogenesis, with the common factor being infection with the oral pathobiont *P. gingivalis*. This pathway, as activated in CP patients, and in mice orally infected with *P. gingivalis* is a common pathway involved in oncogenesis. Thus, our results collectively support a two-hit process, involving Mfa1-fimbriae-DC-SIGN mediated evasion of immune response, and FimA-CXCR4 mediated inflammation and cancer development. Manipulation of this pathway may be therapeutically beneficial for patients in the management of dysbiosis-related immunological disorders as in chronic periodontitis and in preventing cancer development.

## Materials and Methods

### Antibodies and reagents

Anti-human CD4^+^, CD8^+^, CD11b, CD15, CD16, CD33, HLA-ABC and HLA-DR was procured from eBioscience. CD83, CD14, CD209 and CD1c were from eBioscience & Miltenyi Biotech. Anti-FOXO1 mAb (C29H4), Anti-rabbit IgG-HRP and Rabbit (DA1E) isotype control were from Cell signaling. Anti-Bim pAb (PA5–20089) and β-actin (MA1-91399) were obtained from Thermo Scientific. Anti-IDO1 was from LSBio LifeSpan BioSciences. Mouse anti-Akt1 (558316), HRP goat anti-mouse IgG (554002) and Rabbit polyclonal IgG (27478) were from BD Pharmingen. Mouse monoclonal SDF-1 (P-159X) antibody (sc-74271) was from Santa Cruz Biotechnology, Inc. The goat anti-mouse IgG H&L (HRP), Anti-CXCR4 (ab124824) and anti-FOXP3 pAb (ab10563) were procured from Abcam. Sheep anti-mouse IgG (whole molecule, A3563) was from Sigma Aldrich. The antagonists for AKT1 (MK-2206 2HCl) and FOXO1 (AS1842856) were from Selleckchem and Calbiochem, respectively.

### Generation and phenotypic characterization of MoDCs

The conventional MoDCs were generated *in-vitro* as described elsewhere^41^ with minor modifications. Briefly, Human monocytes were isolated from mononuclear fractions of peripheral human blood by negative selection (EasySep™ Human Monocyte Isolation Kit, STEMCELL Technologies Inc. Vancouver, Canada). Cells were seeded in the presence of granulocyte–macrophage colony-stimulating factor (GM-CSF)/interleukin-4 (IL-4) (1000-unit ml^−1^) (Gemini Bio-Products, West Sacramento, CA, USA) at a concentration of 3–4 × 10^5^ cells ml^−1^ in RPMI 1640 (HyClone Laboratories, Logan, Utah, USA) containing 10% heat inactivated FBS (Atlanta Biologicals, Flowery Branch, GA, USA) and antibiotic/antimycotic (HyClone, Logan, UT, USA) for 5–6 days. Fresh medium treated with GM-CSF and IL-4 was used every other day for cultured cells. Flow cytometry analyses were carried out to verify the immature DC phenotype (CD1a^+^CD83^−^CD14^−^DC-SIGN^+^)^15,34,44–46^.

### Flow cytometry

Flow cytometry gates were chosen using suspension cell forward and side scatter characteristics as described^34,46^. Briefly, non-adherent MoDCs typically fall within FSC-h 300–600 and SSC-h 100–200 (x1000). Undifferentiated monocytes, lymphocyte carry-over, and debris were excluded from MoDC analysis. Monocytes displayed 95–99% differentiation into MoDCs by day 5 as described above. Monocytes were immediately infected with all isogenic *P. gingivalis* mutants (DPG3, MFI, MFB) and wild type at 1 MOI^44^ and incubated for 12 hours to generate MDDSCs. DC-SIGN^+^CD1c^+^ cells were chosen for analysis of MDDSCs, CD4^+^ cells for analysis of effector subsets, and CD8^+^ for analysis of CTLs. For statistical analyses, all experimental samples were compared to each other using ANOVA on the means from at least three independent flow experiments using GraphPad Prism6.

### Bacterial culture, labeling, and MoDCs infection

Wild-type *P. gingivalis*381 (*Pg*381), which expresses minor (Mfa1) and major (FimA) fimbriae, isogenic major fimbria-deficient mutant *Pg*-DPG3 (DPG3), which expresses only the minor fimbriae (Mfa1^+^/FimA^−^), the isogenic minor fimbria-deficient mutant *Pg*-MFI (MFI), which expresses only the major fimbriae (Mfa1^−^/FimA^+^) and the double fimbriae mutant/naked (Mfa1^−^/FimA^−^) *Pg*-MFB (MFB) were maintained anaerobically (10% H_2_, 10% CO_2_ and 80% N_2_) in a Coy Laboratory vinyl anaerobic system glove box at 37 °C in Difco anaerobe broth MIC^41^. Erythromycin (5 µg/ml) and tetracycline (2 µg/ml) were added as per the selection requirements of the strains^44,76^. The MoDC were pulsed with *P. gingivalis* strains at multiplicity of infection (MOI) = 1 for 12 hours. The low MOI values were used to mimic a natural blood mDC infection observed in CP patients^15^ as well as to avoid overwhelming the host response.

### RNA sequencing and functional genomics analysis

RNA-sequencing and bioinformatics analysis was performed as we described previously^41^. We have made further analysis for functional cluster, particularly FimA^-^/Mfa1^+^ mutant strain DPG3 due to its greater virulence and *Pg381* (since it expresses both FimA^+^/Mfa1^+^) in DCs relative to other mutants compared with uninfected control and MFB. In brief, each comparison, a gene is defined as differentially expressed using a fold change of ≥±2, average Fragments Per Kilobase of transcript per Million mapped reads (FPKM) ≥ 5 and *P*-value ≤ 0.01. Median FPKM values are used, if a gene has multiple isoforms. Clustering and visualization of differentially expressed transcripts as heatmaps were generated using heatmaps2 for 27 filtered GO terms and annotated genes. The functionality of the selected genes was confirmed by qPCR, Immunofluorescence, Immunoblot and chromatin immunoprecipitation (ChiP) assays. Gene Expression Omnibus (GEO) accession ID #: GSE67141.

### Oral infection with *Pg*381 and fimbriae mutants in acute and chronic murine model of CP

All mice had C57BL/6 backgrounds were purchased from Jackson (Bar Harbor, ME). To determine the immunobiological consequences of oral infection with *Pg*381 and fimbriae mutants (DPG3, MFI and MFB) *in-vivo* (for both acute & chronic), we orally-infected B6 mice (n = 36) with these strains and performed gene expression profiles of isolated blood and splenocytes. Mice were fed a normal chow diet. Six-week old male mice were treated with a 10-day regimen of oral antibiotics (sulfamethoxazole/trimethoprim suspension 48 mg/mL) to allow for *P. gingivalis* colonization. Mice were challenged by oral application of vehicle (2% carboxymethylcellulose-CMC in PBS) or the *P. gingivalis* strains (1 × 10^9^ Colony Forming Units - CFU) at the buccal surface of the maxillary vestibule 5 times a week for 3 weeks as described^77^. The isogenic fimbriae deficient mutants (Table S1) were maintained under proper antibiotic presence to maintain mutant traits as described earlier^44,76,78,79^. Liquid cultures were maintained until mid to late log phase. Bacteria were washed/re-suspended in PBS. 1:10 dilution of the culture and matched to an optical density of 1.0 at 660 nm (O.D. 660 = 1 × 10^9^ CFU/ml). 2% CMC was added slowly to the bacterial suspension while vortexing to avoid clumping. Purity of the cultures was verified by Gram stain before introducing bacteria into animals. Mice were then sacrificed at 1, 12 and 24 hours, and then blood and spleens were collected for RNA isolation (Qiagen). After 4 weeks of chronic infection, mice were sacrificed and mandibles (with attached gingiva) were harvested and fixed in 4% PFA. After 3 days, samples were decalcified with EDTA 14% solution for 3 weeks, then embedded in paraffin and processed for H&E and immunostaining.

### Antibody Analysis

We next determined differences in anti-*P. gingivalis* antibody titers in BL6 mice (n = 12) after oral infection using standard enzyme-linked immunosorbent assay (ELISA) protocol. Briefly, diluted mice sera (1:100) were reacted with the bacterial antigen for 2 hours, then washed and labeled with a secondary antibody goat anti-mouse IgG conjugated to alkaline phosphatase (1:5000). Assays were developed with *p*-nitrophenolphosphate and reactions were terminated by the addition of 3 M NaOH and the optical density (OD) was measured at OD_405_ nm using a BioRad Microplate Reader. Mice were monitored twice a week for clinical symptoms and general health status.

### Cell Proliferation and Inhibitory Assay

Cell proliferation assay was performed as described^48^ with a few modifications, briefly, human head and neck oral squamous cell carcinoma (OSCCs) derived cell lines, HN6 and HN12 (a gift from Dr. Yeudall AW, Department of Oral Biology, AU, Augusta, GA, USA) were cultured as described^80^ and seeded in 6-well plates at 1–5 × 10^5^ cells per well in the growth medium. Cells were uninfected or incubated with DPG3, MFI, *Fad*A^+^ and LPS (100 ng/ml) (as positive controls) or inhibitors added at the concentration of AMD3100 (50 nM), MK-2206 (2 µM), AS1842856 (50 nM), gp120 (6 µg/ml) prior to co-culture with *P. gingivalis* at MOI = 1. The concentrations of inhibitors (CXCR4 inhibitor AMD3100; AKT inhibitor MK-2206; FOXO1 inhibitor AS1842856) selected based on the IC_50_ value according to the manufacturer’s instruction. Cell numbers were counted at 0, 24, 48 and 72 hours using a hemocytometer. Human tonsillar keratinocytes hTERT HAK Clone 41 (a gift from Dr. A. Klingelhutz and Dr. J. Lee, U. Iowa, Iowa City, IA, USA^81^ were cultured in KSFM with 0.2 ng/ml epidermal growth factor (EGF) and 30 μg/ml bovine pituitary extract. Previous studies showed that these telomerase-immortalized keratinocytes (hitherto labeled ‘tertAd7cl41’) were functionally similar to primary oral keratinocytes^82^. The proliferation assay done as described above. Each experiment was performed in triplicate and repeated thrice.

### Gingival biopsies and blood collection from clinically healthy subjects and CP patients

The study was conducted between December 2013 and May 2016 in full accordance with the Helsinki Declaration of 1975, as revised in 2000. All the patients were recruited from the School of Dentistry, University of São Paulo and Dental College of Georgia AU. A detailed medical and dental history was obtained from all patients. All volunteers received full mouth periodontal clinical examination performed at six sites per tooth (excluding third molars). CP subjects with moderate to severe disease, determined by exhibition of the following: probing depth of >4 mm, attachment loss of >3 mm, bleeding on probing, and alveolar bone crest >3 mm from the cementoenamel junction. Gingival tissue biopsy and blood samples from CP patients (n = 24) and healthy controls (n = 25) were collected. We have previously reported our biopsy technique to obtain oral lymphoid foci from the interdental papilla without removing buccal and lingual epithelium at the deepest bleeding site^83,84^. These tissues were cryosectioned in OCT for H&E and immunofluorescence analysis by confocal microscopy. Blood was also collected for transcriptome and DC subset analyses as described above.

### TaqMan-Array and SYBR-Green qPCR Assays

Analysis of gene expression in MDDSCs induced by *Pg*381, DPG3, MFI, and MFB, MoDCs and monocytes was performed using RT-PCR. Total RNA was isolated using RNeasy kit from three groups such as (1) *In-vitro* control and *P. gingivalis* strains at 1 MOI infected monocytes were cultured for 6 hours, and day 6 MoDCs; (2) *In-vivo* mice blood and spleens at 1, 12 and 24 hours; and (3) *Ex-vivo* human blood panDCs isolated from healthy control and chronic periodontitis patients. Human blood DCs were negatively selected from PBMCs using panDC pre-enrichment kit (Robosep, Stem cell technologies). The isolation was performed two times to remove non-DCs. The purity of enriched DCs was 70–80%. Complementary DNA was synthesized from 1.1 μg RNA through a reverse-transcription reaction (Applied Biosystems). Real-time quantitative PCR (RT-qPCR) was performed on TaqMan array fast plates as well as reconfirmed with SYBR green methods for selected genes (PCR primers are listed in Tables S2 & S3). The calculations for fold regulation used the 2^−ΔΔCt^ method as described in our previous report^41^. 18SrRNA, HPRT1 and GAPDH were used as internal controls.

### Immunofluorescence Assay (IFA)

IFA was performed for paraffin embedded tissues. Briefly, the paraffin embedded tissues from mice (CMC, *Pg*381 and DPG3 oral infected CP model) and human (healthy and CP patients) were rehydrated and antigen retrieved. After blocking with permeabilization buffer and 2% BSA, slides were then incubated with primary antibodies followed by respective secondary antibodies. Slides were mounted with prolong diamond anti-fading mounting medium (Invitrogen). Images were acquired with a Zeiss LSM510 Inverted Meta scanning confocal microscope. For quantitative analysis, four fields were selected randomly and total cells in the field were manually counted based on their expression of the target proteins.

### Immunoblot and Receptors Blockade Assays

Immature MoDC were infected with WT-*Pg*381, DPG3 and MFI for 6 hours at 1 MOI. DC-SIGN was blocked with GP120 (6ug/ml) for 30 minutes before infection. Likewise, OSCCs were infected with *Pg*381, DPG3, MFI, *Fad*A^+^ at MOI = 1 and LPS (100 ng/ml) (both *Fad*A^+^ and LPS used as positive controls). CXCR4, pAKT, pFOXO1 and DC-SIGN were blocked with their respective antagonist (inhibitors) at the concentration of AMD3100 (50 nM), MK-2206 (2 µM), AS1842856 (50 nM), and gp120 (6 µg/ml). The concentrations of inhibitors were selected based on the IC_50_ value as per the manufacturer’s instruction. Whole-cell lysates were prepared from both MoDCs and OSCCs cultures by centrifuging and washing the pellet with ice-cold PBS. The gingival tissue used for this study was obtained from chronic periodontitis (CP) subjects (generalized severe) and healthy adult controls as previously described^83^. Proteins from human gingival tissues, mouse spleen and the cells were extracted by adding ice-cold Radioimmunoprecipitation assay (RIPA) buffer (Abcam) supplemented by protease inhibitor cocktail (Cell signaling). The cell lysates were centrifuged at 12,000 × g for 10 min at 4 °C. Samples were normalized to the amount of total protein in the supernatant using Pierce™ BCA Protein Assay Kit (ThermoFisher). Protein aliquots (30 μg) were separated by size on 4–15% Mini-PROTEAN TGX stain-free precast gels (Bio-Rad Laboratories, Inc.) and transferred to Immobilon (PVDF) membrane (EMD Millipore). Nonspecific binding sites were blocked by incubation in 1 × TBS-T (0.2 m Tris, 0.14 m NaCl, 0.1% Tween 20) containing 5% non-fat milk for 1 hour at room temperature, followed by incubation overnight at 4 °C with 1:1000 dilution of primary antibodies (Akt, p-Akt, FOXO1, p-FOXO1, DC-SIGN (CD209), and GAPDH) in 1 × TBS-T containing 5% non-fat milk. After membranes were washed three times in 1 × TBS-T (10 min each at room temperature), horseradish peroxidase-conjugated secondary antibody was added at a 1:2000 dilution and incubated for 1 hour at room temperature. After three more washes with 1 × TBS-T, the immunoreactive peptide was detected by Western Lightning ECL Pro Chemiluminescent reagent (PerkinElmer, Inc) and imaged using ChemiDoc™ MP Imaging System (Laboratories, Inc.). Data were analyzed by performing one-way ANOVA with Tukey’s post hoc using Prism GraphPad.

### Chromatin Immunoprecipitation (ChIP)

ChIP analyses were done as described^36^. Briefly, once the unicellular suspension was obtained from the gingival tissues of CP patients and healthy subjects, cells (5 × 10^6^) were fixed for 10 min at 25 °C with 10% formaldehyde. After incubation, glycine was added to a final concentration of 0.125 M to ‘quench’ the formaldehyde. Cells were pelleted, washed twice with ice-cold PBS and lysed. The lysates were pelleted, re-suspended and sonicated to reduce DNA length to 300–500 base pairs. Chromatin prepared (#D5210, Zymo Research Corporation, CA) from CP patients and healthy control gingival tissue and cells were pre-cleared for 1 hour with protein Ultralink A/G agarose beads (Pierce, #88803) and then was incubated overnight with 2 µg ChIP grade anti-AKT1 (MA5–14898; ThermoFisher), or anti-FOXO1A (ab39670; Abcam), or control rabbit immunoglobulin (2729; Cell Signaling), and one without antibody as the input. Particle Concentrator (#123.21D, Invitrogen), washed, and eluted in 100 µl of 0.1 M NaHCO3 and 1% SDS elution buffer. The eluted and input chromatins were incubated at 65 °C (for 3 hours) to reverse the crosslinking. After digestion with proteinase K, the ChIP and input DNA were purified (using Chip DNA Clean & Concentrator Kit, #D5205 Zymo Research) and analyzed by quantitative PCR using site-specific primers binding of AKT1 with the co-factors to the FOXO1 and FOXO1 to the FOXP3 and BIM locus (Table S4).

### Ethical approval and informed consent

The Human Assurance Committee (HAC) at the Augusta University approved this protocol involving human cells as human subject exempt, due to the use of anonymized peripheral blood samples for monocytes. The study protocol and informed written consent form obtained from all subjects were reviewed and approved by the School of Dentistry Ethics Committee, University of São Paulo, Brazil (658.998/CEP), and the Augusta University IRB (831204–3). All procedures involving mice were reviewed and approved by the Augusta University Institutional Animal Care and Use Committee (IACUC ID: # 2013-0586)/ethics committee and were performed in accordance with the approved guidelines and regulations.

## Electronic supplementary material

Supplementary Information

## Data Availability

The datasets generated and analyzed during the current study are available from the corresponding authors on reasonable request.
