# Supplementary material for: Oral Pathobiont Activates Anti-Apoptotic Pathway, Promoting both Immune Suppression and Oncogenic Cell Proliferation
Source: Sci Rep. 2018 Nov 9;8:16607. doi: 10.1038/s41598-018-35126-8 (PMC6226501; doi:10.1038/s41598-018-35126-8)
Supplement: Supplementary file 1 — Supplementary Information [file 41598_2018_35126_MOESM1_ESM.pdf]

Oral Pathobiont Activates Anti-Apoptotic Pathway, Promoting both Immune Suppression and Oncogenic Cell Proliferation

Pachiappan Arjunan[1¶*], Mohamed M. Meghil[1,3¶], Wenhu Pi[2], Jinxian Xu[1], Liwei Lang[3], Ahmed El-Awady[1], William Sullivan[4], Mythilypriya Rajendran[1], Mariana Sousa Rabelo[1,5], Tong, Wang[1], Omnia K. Tawfik[1] Govindarajan Kunde-Ramamoorthy[6], Nagendra Singh[7], Thangaraju Muthusamy[7], Cristiano Susin[1], Yong Teng[3], Roger M. Arce[1], and Christopher W. Cutler[1*]

[1]Department of Periodontics, Dental College of Georgia, Augusta University, Augusta, Georgia, United States of America. [2]Department of Radiation Oncology, Indiana University, Indianapolis, Indiana, United States of America. [3]Department of Oral Biology, Augusta University, Augusta, Georgia, United States of America. [4]Department of Energy, Joint Genome Institute, California, United States of America. [5]Department of Periodontics, University of São Paulo, Brazil. [6]The Jackson Laboratory for Genomic Medicine, Connecticut, United States of America. [7]Department of Biochemistry & Molecular Biology, Cancer Research Center, Augusta University, Augusta, Georgia, United States of America.

Condensed title: Chronic Periodontitis Microbe Promotes oncogenic cell proliferation

¶Equal Contribution

[*]**Corresponding authors**
Christopher Cutler, DDS, PhD
Phone #: 706-721-2442; E-mail: chcutler@augusta.edu
Department of Periodontics, Dental College of Georgia, Augusta University, Augusta, Georgia, USA.

Pachiappan Arjunan, PhD
Phone #: 706-446-5176; E-mail: parjunan@augusta.edu
Department of Periodontics, Dental College of Georgia, Augusta University, Augusta, Georgia, USA.

Supplementary Information

SI Figure S1. Transcriptome and phenotype indicates non-canonical DCs are MDDSCs not MDSCs. Monocytes were isolated from three healthy individuals and infected with DPG3 strains for 12 hours. At 0 and 12 hours cells were isolated and stained with antibodies for flow cytometry and RNA isolated for reverse transcription PCR (RT-PCR). The fold regulation of the gene in each group was calculated relative to its expression in the control samples for that gene and calculated by using HPRT1 and GAPDH reference expression. (A) Log 2- fold differences in gene expression of MoDCs and MDDSCs normalized against control monocytes are plotted against p values for t-tests. Each point in the volcano plot represents the average fold regulation from 3 arrays. All values above the dotted line ($P<0.05$) indicate differences that were significant. (B) Histograms show the surface expression of MDSCs markers on MDDSCs and control monocytes (MoDCs) by flow cytometry. The expression was calculated based on the isotype controls (red). Data are from one representative of four independent experiments. $^{**}P\leq0.01$, $^{***}P\leq0.001$.

SI Figure S2. Transcriptional and functional profiling reveals angio-/oncogenic, anti-apoptotic, immunosuppressive, immuno- regulatory MDDSCs. Monocytes cultured in presence of GM-CSF and IL-4 for 6 days to generate MoDCs or 12 hours with *Pg*-WT (*Pg*381) and DPG3 at 1:1 MOI to generate MDDSCs. (A) RNA-sequencing functional analysis shows the expression of DC-signature genes in response to *Pg381* and DPG3 (refer methods) compared with uninfected control, partitioned into 5 clusters as shown. (B) TaqMan qPCR of transcriptome of MoDCs induced by DPG3 relative to uninfected control (MoDCs) and confirmed by SYBR-qPCR. Fold-change in gene expression was normalized to control monocytes and ≥±2 fold was

considered significant ($P<0.05$). All markers were designed in triplicates on array plates. (C). Immunoblot analysis of apoptotic protein BIM in control MoDCs, induced by *Pg*381, DPG3 and MFI. Results are representative of three independent experiments and refer Fig. 1F for quantification analysis.

SI Figure S3. Distinct immune markers in murine blood after *Pg*381 and its mutant DPG3 oral acute infection. (A-C) The gene expression profiles show the differential response of blood isolated from *Pg*381, DPG3- and MFI-infected mice at three different time (1, 12, 24 hours) points. A negative control group (2% CMC no bacteria) was included. In total 36 mice were then sacrificed at 1, 12 and 24 hours from 4 different groups (refer Fig. 2) and blood and splenocytes collected for TaqMan® array. Results are representative of 18 experiments (n=3 mice/time point).

SI Figure S4. Increased expression of pAKT1 in gingiva, pFoxo1 and Foxp3 proteins in spleen of chronic periodontitis murine model. (A) Immunofluorescence analysis (IFA) of pAkt1 protein expression in gingival interproximal papilla area between M1&M2 from DPG3, *Pg*381 infected mice, compared with uninfected CMC control group. (B) Immunostaining of pFoxo1 and Foxp3 protein expression in spleen tissue from DPG3, *Pg*381 orally infected mice for 4 weeks, compared with CMC control. Images are representative of 3 independent experiments (Scale Bar: - 20μm). Note:- This is the image representing independent channels with respect to Figure 2D.

SI Figure S5. Wild-type *Pg*381 (*Pg*381) and the FimA not significantly stimulated the proliferation of noncancerous human epithelial (ARPE) and mouse fibroblast (MEF) cells, compared to uninfected cells. *Pg*381 strains and also FadA+ were not stimulating or aggravating the proliferation of noncancerous cells, however, apoptotic cell deaths were observed, particularly at 48 and 73 hours in human AEPE cells compared with untreated cells.

SI Figure S6. Increased expression of DC-SIGN, pAKT1, pFOXO1 and FOXP3 in gingival tissue of CP patients. Co-localized expression of pFOXO1 & pAKT1 (A), DC-SIGN & pAKT1 (B) (arrowhead) and DC-SIGN & FOXP3 in gingival connective tissue (marked in Fig. 4B) from CP, compared with healthy control. (D-F) Quantification of co-localization among pFOXO1, pAKT, DC-SIGN and FOXP3 in human gingival tissue. Images are representative of three independent experiments (Scale bar- 100µm).

SI Figure S7. Approaches used to show the mechanistic role of *P. gingivalis*, its fimbriae in immune suppression and oncogenic cell proliferation.

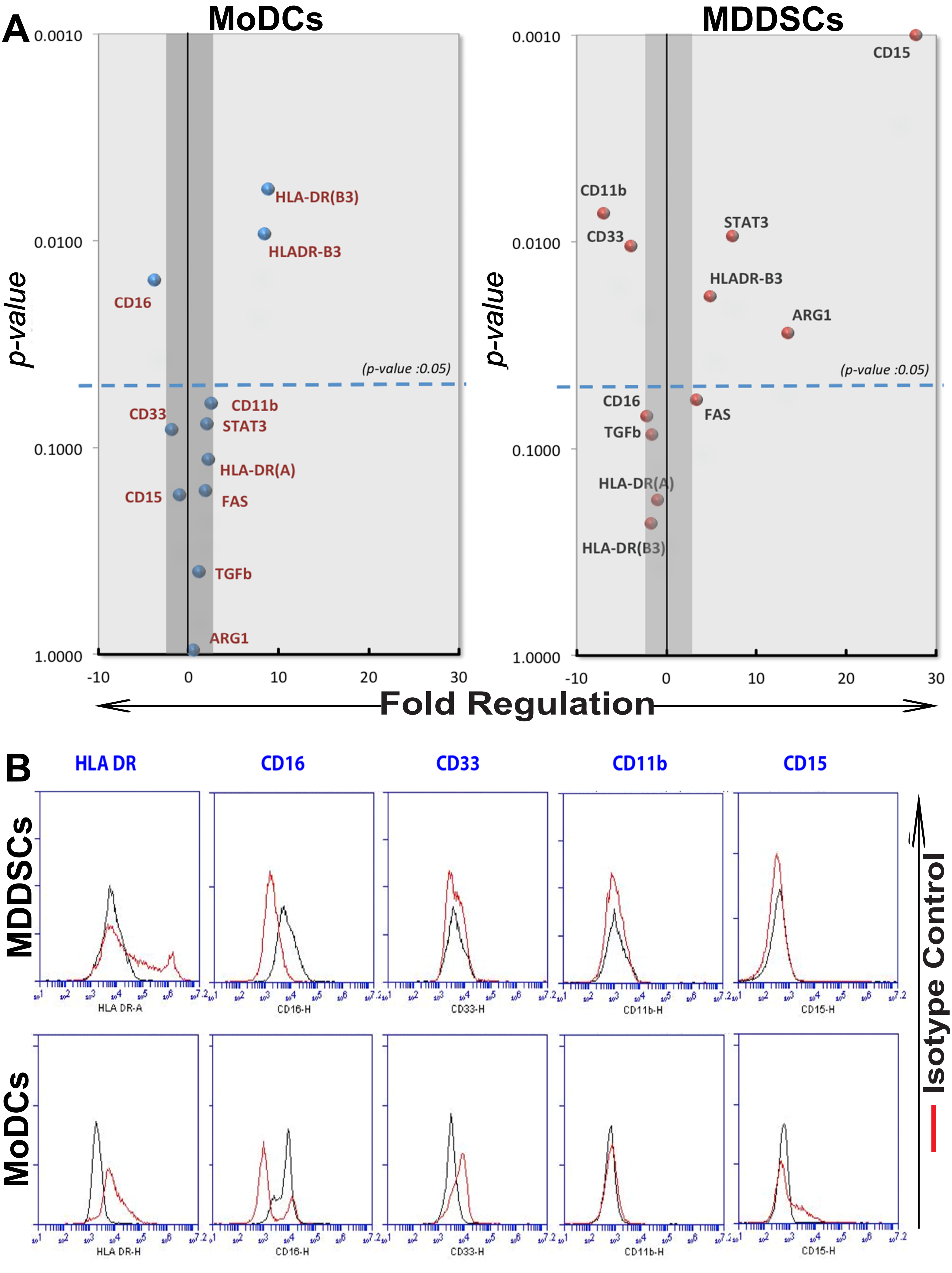
A
MoDCs
MDDSCs
p-value
0.0010
0.0100
0.1000
1.0000
HLA-DR(B3)
HLADR-B3
CD16
(p-value :0.05)
CD11b
CD33
STAT3
HLA-DR(A)
CD15
FAS
TGFb
ARG1
-10
0
10
20
30
Fold Regulation
B
HLA DR
CD16
CD33
CD11b
CD15
Isotype Control
HLA DR-A
CD16-H
CD33-H
CD11b-H
CD15-H
HLA DR-H

Figure S1.

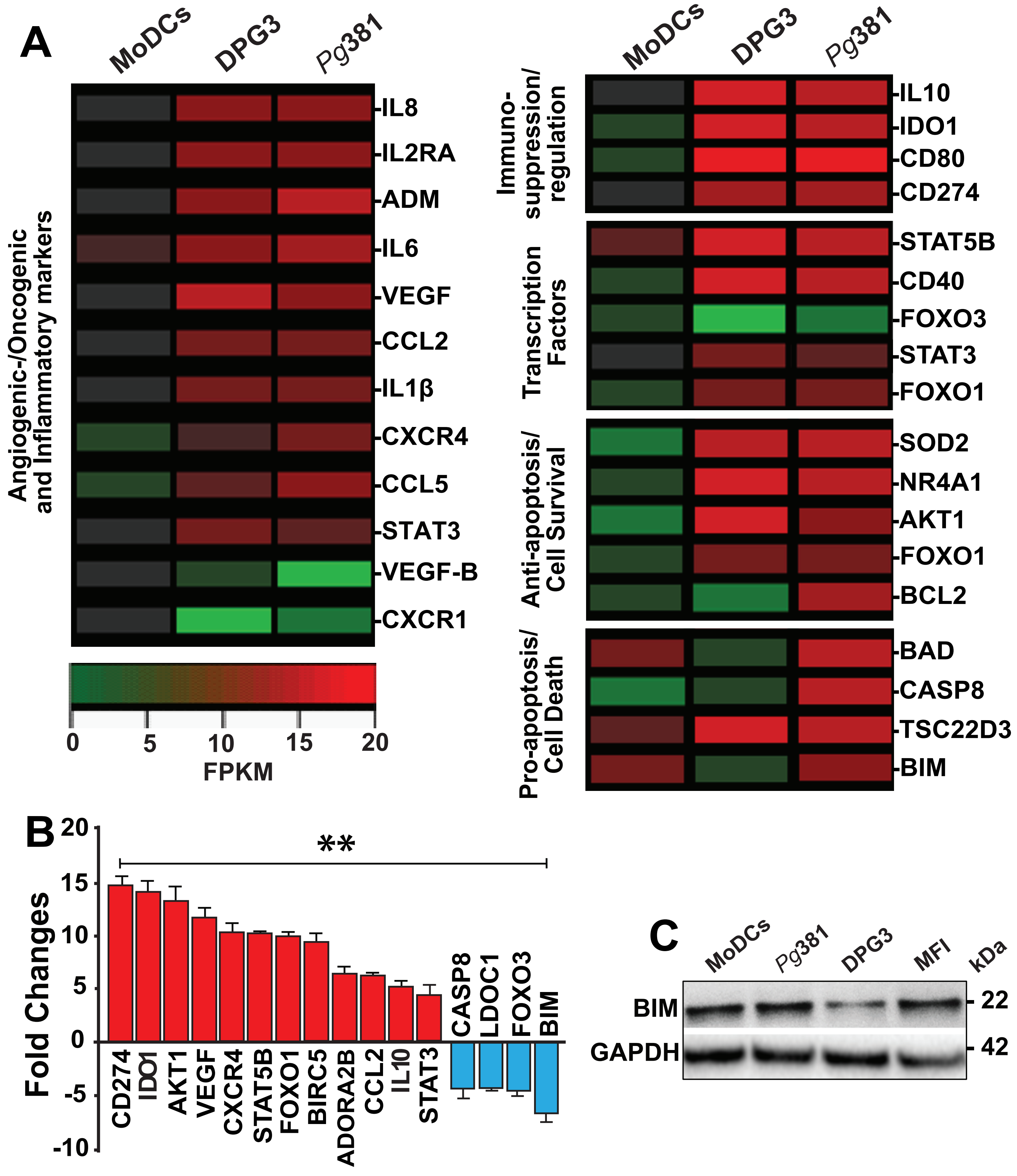
A
MoDCs
DPG3
Pg381
IL8
IL2RA
ADM
IL6
VEGF
CCL2
IL1β
CXCR4
CCL5
STAT3
VEGF-B
CXCR1
Angiogenic-/Oncogenic and Inflammatory markers
0
5
10
15
20
FPKM
Immuno-suppression/regulation
IL10
IDO1
CD80
CD274
Transcription Factors
STAT5B
CD40
FOXO3
STAT3
FOXO1
Anti-apoptosis/Cell Survival
SOD2
NR4A1
AKT1
FOXO1
BCL2
Pro-apoptosis/Cell Death
BAD
CASP8
TSC22D3
BIM
B
Fold Changes
20
15
10
5
0
-5
-10
**
CD274
IDO1
AKT1
VEGF
CXCR4
STAT5B
FOXO1
BIRC5
ADORA2B
CCL2
IL10
STAT3
CASP8
LDOC1
FOXO3
BIM
C
MoDCs
Pg381
DPG3
MFI
kDa
BIM
GAPDH
22
42

Figure S2

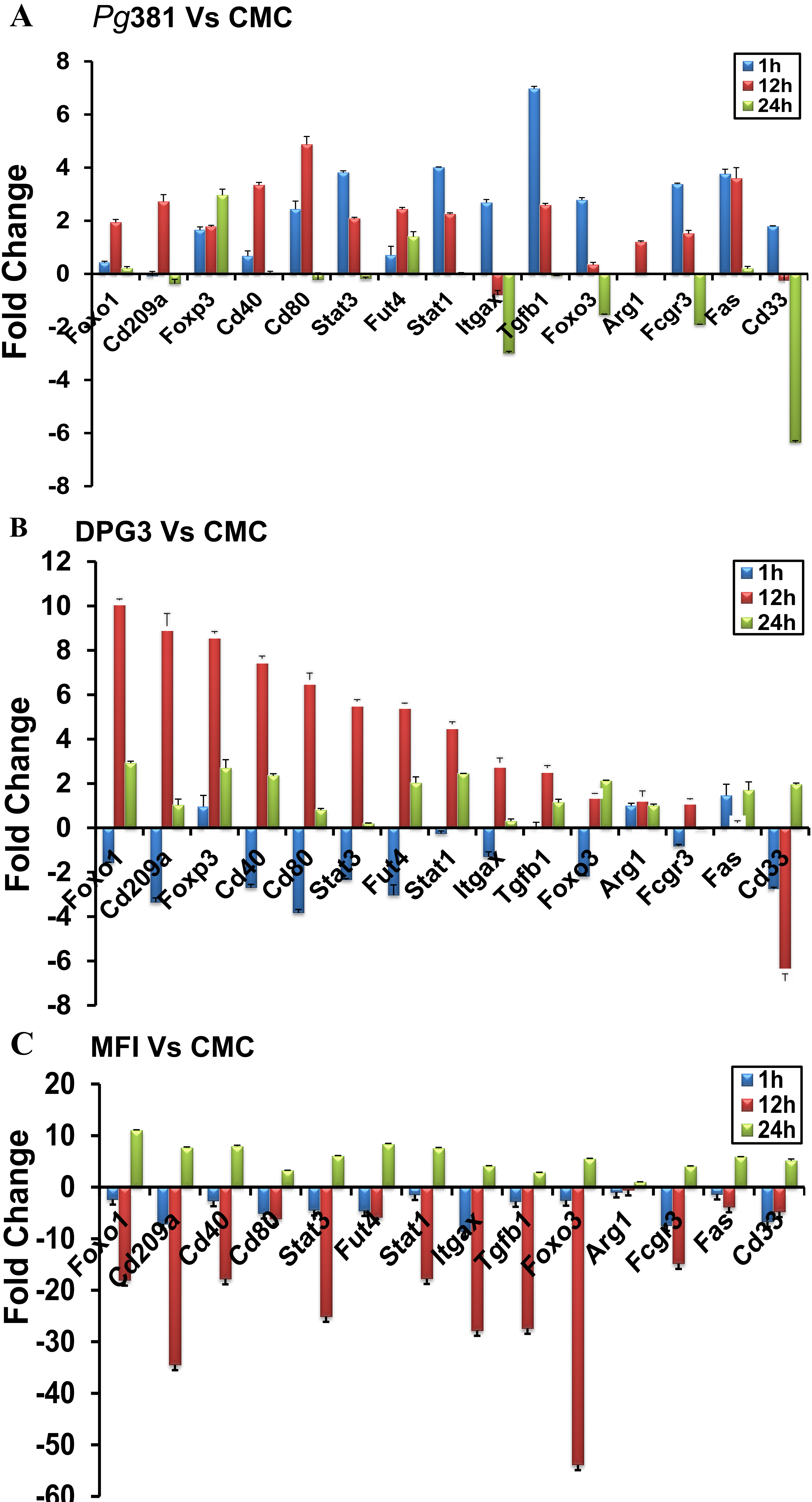
A
*Pg*381 Vs CMC
Fold Change
8
6
4
2
0
-2
-4
-6
-8
1h
12h
24h
Foxo1
Cd209a
Foxp3
Cd40
Cd80
Stat3
Fut4
Stat1
Itgax
Tgfb1
Foxo3
Arg1
Fcgr3
Fas
Cd33
B
DPG3 Vs CMC
Fold Change
12
10
8
6
4
2
0
-2
-4
-6
-8
1h
12h
24h
Foxo1
Cd209a
Foxp3
Cd40
Cd80
Stat3
Fut4
Stat1
Itgax
Tgfb1
Foxo3
Arg1
Fcgr3
Fas
Cd33
C
MFI Vs CMC
Fold Change
20
10
0
-10
-20
-30
-40
-50
-60
1h
12h
24h
Foxo1
Cd209a
Cd40
Cd80
Stat3
Fut4
Stat1
Itgax
Tgfb1
Foxo3
Arg1
Fcgr3
Fas
Cd33

Figure S3

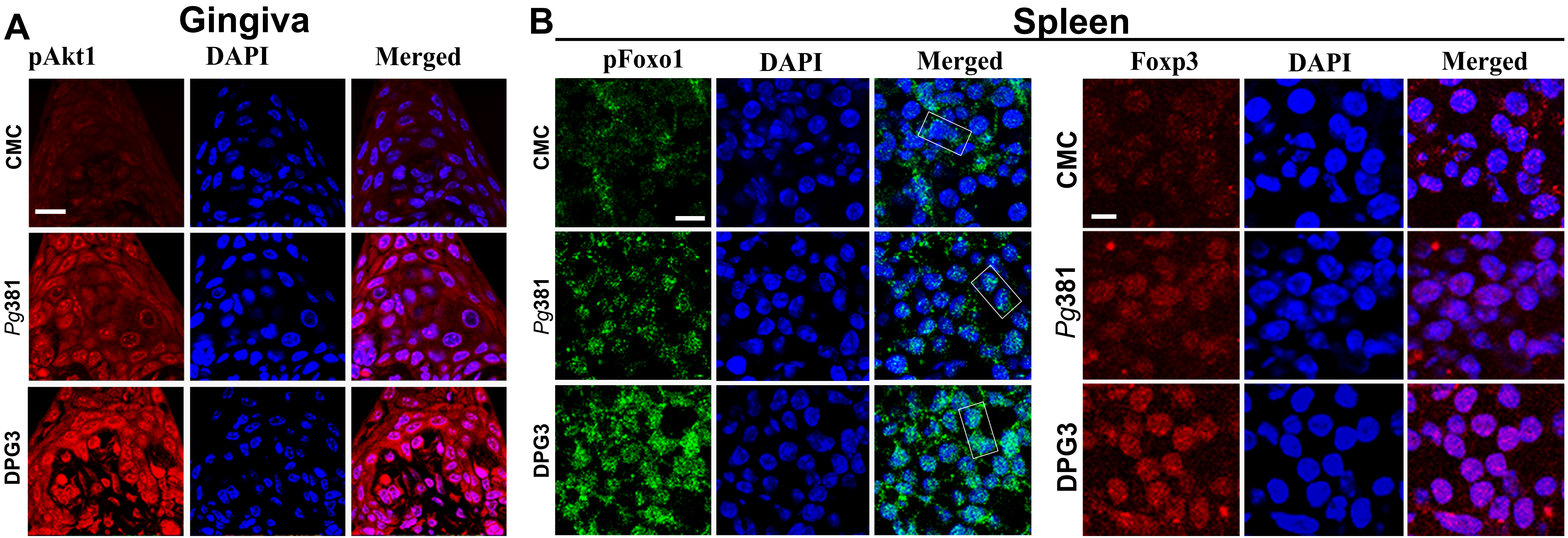

A
Gingiva
pAkt1
DAPI
Merged
CMC
Pg381
DPG3
B
Spleen
pFoxo1
DAPI
Merged
Foxp3
DAPI
Merged
CMC
Pg381
DPG3

Figure S4

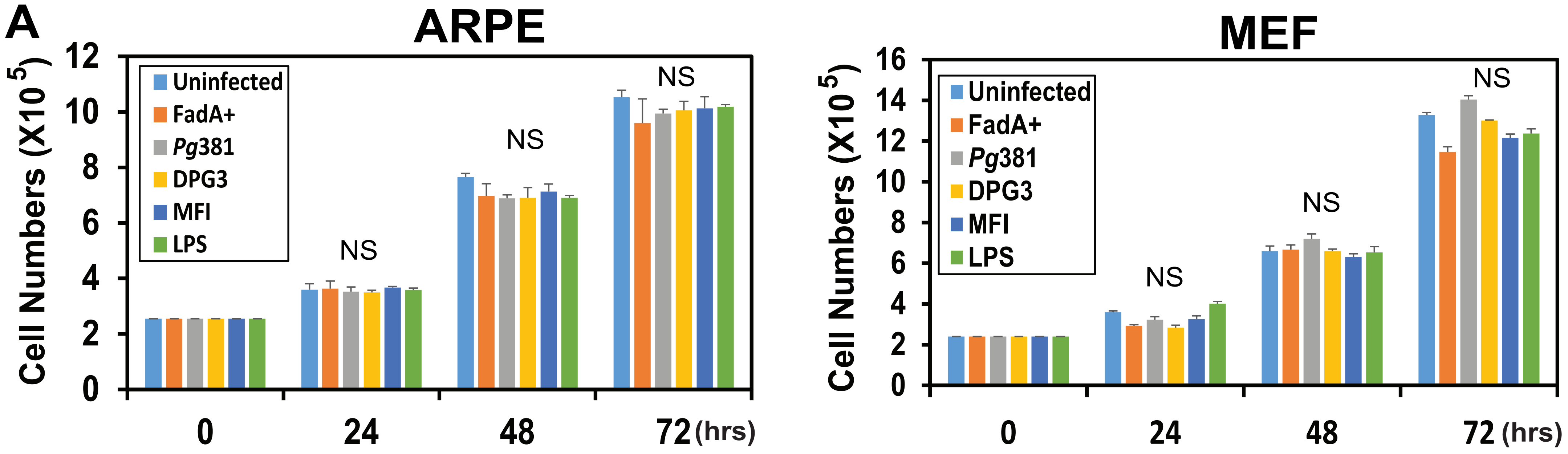
A
ARPE
Cell Numbers (X10^5)
12
10
8
6
4
2
0
Uninfected
FadA+
Pg381
DPG3
MFI
LPS
NS
NS
NS
0
24
48
72 (hrs)
MEF
Cell Numbers (X10^5)
16
14
12
10
8
6
4
2
0
Uninfected
FadA+
Pg381
DPG3
MFI
LPS
NS
NS
NS
0
24
48
72 (hrs)

Figure S5

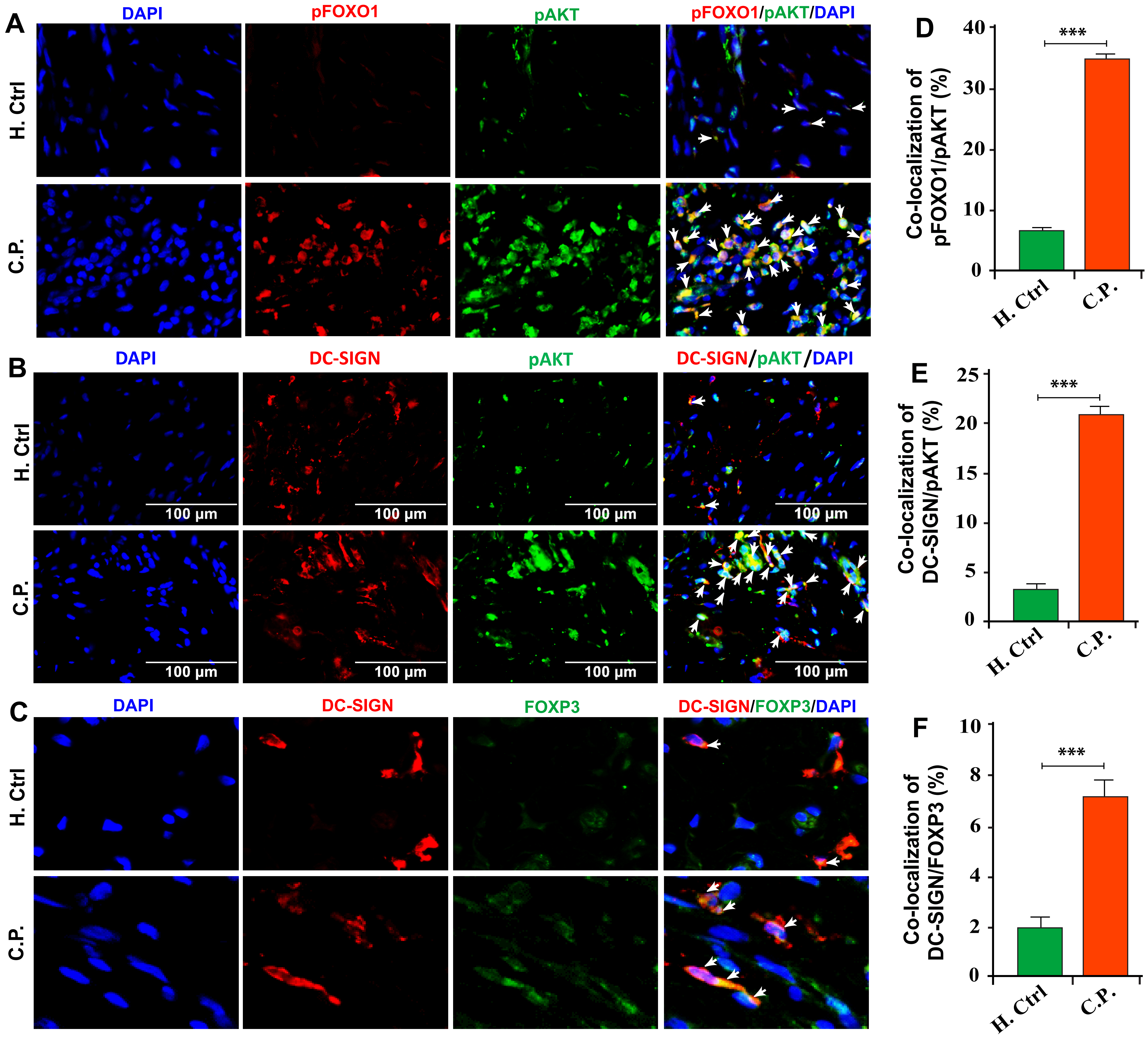
A
B
C
D
E
F
DAPI
pFOXO1
pAKT
pFOXO1/pAKT/DAPI
DC-SIGN
DC-SIGN/pAKT/DAPI
FOXP3
DC-SIGN/FOXP3/DAPI
H. Ctrl
C.P.
100 μm
Co-localization of pFOXO1/pAKT (%)
Co-localization of DC-SIGN/pAKT (%)
Co-localization of DC-SIGN/FOXP3 (%)
***

Figure S6

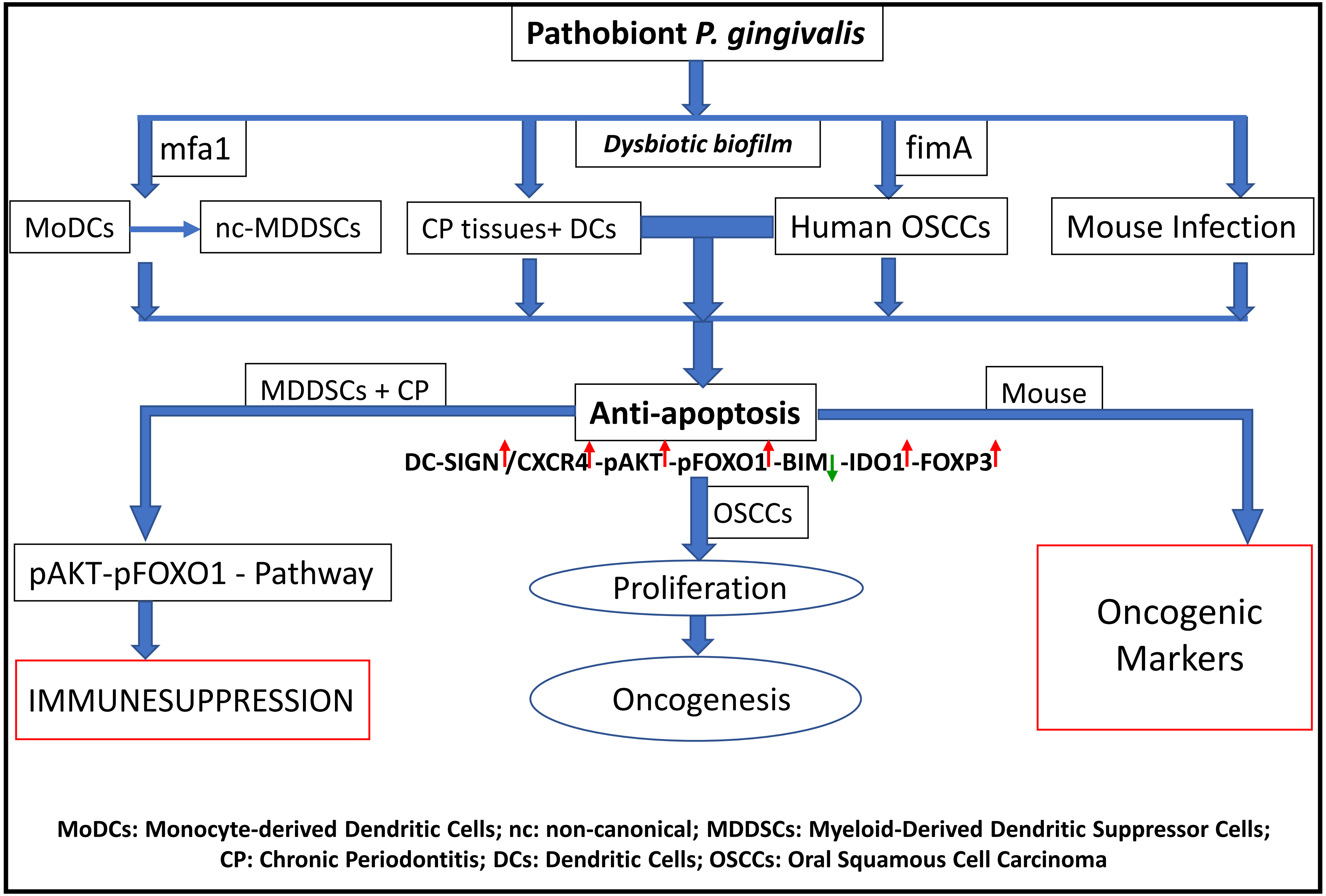
Pathobiont P. gingivalis
mfa1
Dysbiotic biofilm
fimA
MoDCs
nc-MDDSCs
CP tissues+ DCs
Human OSCCs
Mouse Infection
MDDSCs + CP
Anti-apoptosis
Mouse
DC-SIGN↑/CXCR4↑-pAKT↑-pFOXO1↑-BIM↓-IDO1↑-FOXP3↑
OSCCs
pAKT-pFOXO1 - Pathway
Proliferation
Oncogenic Markers
IMMUNESUPPRESSION
Oncogenesis
MoDCs: Monocyte-derived Dendritic Cells; nc: non-canonical; MDDSCs: Myeloid-Derived Dendritic Suppressor Cells;
CP: Chronic Periodontitis; DCs: Dendritic Cells; OSCCs: Oral Squamous Cell Carcinoma

Figure S7

Supplementary Table S1. ***P.gingivalis*****381 and its isogenic fimbriae mutant strains.**

Description of *P.gingivalis* wild type 381 and its isogenic fimbriae deficient mutant strains[1] used in this study			
Strain	Phenotype[1,2]	Genotype[3]	Predominant PRRs targeted by fimbriae
*Pg*381	Mfa1+/FimA+, modest autophagy evasion, 24hr survival in semi-mature DCs	381	DC-SIGN[4,5]/TLR2[2]-CXCR4[6]
Pg-DPG3	Mfa1+/FimA-, high autophagy evasion, 48hr survival in immature DCs	381ΔfimA	DC-SIGN
Pg-MFI	Mfa1-/FimA+, eliminated by autophagy by 12hr in DCs	381Δmfa1	TLR2-CXCR4

References:

1. Takahashi, Y., et al. (2006). "Fimbria-dependent activation of pro-inflammatory molecules in Porphyromonas gingivalis infected human aortic endothelial cells." Cell Microbiol **8**(5): 738-757.

2. El-Awady, A. R., et al. (2015). "Porphyromonas gingivalis evasion of autophagy and intracellular killing by human myeloid dendritic cells involves DC-SIGN-TLR2 crosstalk." PLoS Pathog **10**(2): e1004647.

3. Arjunan, P., et al. (2016). "High-throughput sequencing reveals key genes and immune homeostatic pathways activated in myeloid dendritic cells by Porphyromonas gingivalis 381 and its fimbrial mutants." Mol Oral Microbiol **31**(1): 78-93

4. Zeituni, A. E., et al. (2009). "Targeting of DC-SIGN on human dendritic cells by minor fimbriated Porphyromonas gingivalis strains elicits a distinct effector T cell response." J Immunol **183**(9): 5694-5704.

5. Zeituni, A. E., et al. (2010). "The native 67-kilodalton minor fimbria of Porphyromonas gingivalis is a novel glycoprotein with DC-SIGN-targeting motifs." J Bacteriol **192**(16): 4103-4110.

6. Hajishengallis, G., et al. (2008). "Pathogen induction of CXCR4/TLR2 cross-talk impairs host defense function." Proc Natl Acad Sci U S A **105**(36): 13532-13537.

Supplementary Table S2. List of primers for SYBR Green-qPCR analysis

Gene Name	GenBank ID	Species	Description	Sense Strand (FW) (5'-3')	Anti-Sense (RV) (5'-3')	Product Size (bp)
FOXO1	NM_002015	Human	Forkhead box O1	TGTCCTACGCCGACCTCAT	TTGAATTCTTCCAGCCCGCC	150
Foxo1	NM_019739	Mouse	Forkhead box O1	GGCGGGCTGGAAGAATTCAA	GCCCTCGGCTCTTAGCAAAT	188
IDO1	NM_002164.5	Human	Indoleamine 2,3-dioxygenase 1	TGGCCAGCTTCGAGAAAGAG	CTTGGCAAGACCTTACGGACA	159
Ido1	NM_008324.2	Mouse	Indoleamine 2,3-dioxygenase 1	CCACATAGATGAAGATGTGGGCT	CAGTGTGGGCAGCTTTTCAAC	151
ARG1	NM_001244438.1	Human	Arginase 1 (ARG1) transcript variant	TGGACCCTGGGGAACACTAC	GGGTCCAGTCCGTCAACATC	169
Arg1	AB047402.1	Mouse	Arginase 1 (Arg1)	CAGGGCAATTGTGCAGGTTC	GGTGGGTATCACAGGACAGC	130
ZNF366	NM_152625.2	Human	Zinc finger protein 366 (ZNF366)	GCCAACATGAAGCGACACG	TCCTTCCCACAAAGCTTGCAT	155
Znf366	NM_001004149.1	Mouse	Zinc finger protein 366 (Znf366)	TCAAGGCTCACATGATCGTCC	CCCCTACCCAGACCTTGAGAG	238
Bim	NM_207680.2	Mouse	BCL2-like 11	GCCAGGCCTTCAACCACTAT	TGCAAACACCCTCCTTGTGT	153
AKT1	NM_001014431.1	Human	AKT serine/ threonine kinase 1	CAGGATGTGGACCAACGTGA	AAGGTGCGTTCGATGACAGT	137
Akt1	NM_001165894.1	Mouse	AKT serine/ threonine kinase 1	CCGCCTGATCAAGTTCTCCT	TTCAGATGATCCATGCGGGG	118
Stat3	NM_213659.3	Mouse	signal transducer and activator of transcription 3	TAAGCAGCCGAACCCCATATC	CCAGACTCAGAGGTGCTCTC	173

Supplementary Table S3. Probe/primer sequences of Mfa1 and FimA for TaqMan qPCR analysis

Gene Name	Assay ID	Probe	Forward Primer	Reverse Primer
FimA	AIY9ZZQ	CTGCGCTCATTTGC	CGCATGGCTTTCACCGAAATTAAA	TCAGGGACGAATGTGTAAATGTTATCG
Mfa1	AIX01UM	TTGGCGACGTTCTCC	GGATATCAGATGGGTTGTTGCTCAA	AACAGTTCCTCTTTTCTTGCTTAGGT
*Pg*16s	AIY9ZZ2	CTACACCACGAATTCC	TGCCGGGCTTGAGTTCAG	GAGTTCCTCGTGATATCTATGCATTTCA

Supplementary Table S4. Transcription binding site for the promoter region and the specific primers

1. FOXO1 gene (Human) promoter

```
-820 ACAGTGAATC CGGCTGGGGG CTGCCGGCTG GGTGACGCGC CTCTGGCTAG ACCGAATGGC
-760 ACAGGCTGAG CCCGGGGGTG CGGACCGGTG GGCACGGAGA AGGGTCCCTC GGCGCGGACT
-700 CGACTGCAGG AGCCGCGGCG CCGGGGGTGG GGGGCGTGTG CCGCTCGCGA GGGGGCCGGA
-640 GGACTTGACA GATCGCAGCG AAAGAAAACA TTAAACCAAA ACAAAACCCA CCGATTCCCC
-580 ACGTCGTTCA GCAAAGACAT CGTGGGTGGA GCCAGGAGGG AGCGATTGAG TAGAATTCCT
-520 CGCGGCCGCC TCCGCCCGCC CACCCCGCGC CCCCGGCCCT CGGCCCCCTG CCCTGCCAGC
-460 CCGCCGGGCT CGGCCCGCAG CGGAGGTCAG GGAGCAGCGA GCGCCTCCTC CCACCCGGGC
                                 FOXO1,3,4,P2
-400 TTCCCGAGTA CTCGGCTCTG CTGCTCCGTA GTAAACAAAG TGTCGCCGCC GCCTCCACGC
-340 TGGTTTGCTT CCTAGCAATC AAAACACTGA GAAGGCGAGA GAATCACAGA AACACTCGAG
               FOXP3
-280 AATTACCAGA AAATAATAGA GATCCAAAAA AAAAAAAAGG AGGTGAGTGT GTGAAAGAGA
-220 AAAACACCCC ACTACCCCCC ACCAGCCCAC CGCCGCCTCC CCGTGGAAAA CCGGGCCCCA
-160 CCCAGCCCGG CGCCCACTGG CTGCCCGGGC GGCGGTGCCG CATGCCCATT GGCCGCGCGG
-100 GCTGTCGGTC AGGGGCGGGC CGGCGCGCGC GCCGCCGCGG GCGGGGGGCG GCGGCAGATC
                                            ↱INS
 -40 CCGTAAGTCG GGCGGCCTGG TAGTCGCAGC AGCCGCTGCC gcagccgcca cattcaacag
 +21 gcagcagcgc agcgggcgcg ccgctgggga gagcaagcgg cccgcggcgt ccgtccgtcc
```

FOXO family binding site:- **TTGTTTAC (GTAAAT/CAA)** [conserved consensus core recognition motif (Furuyama et al., 2000; Xuan and Zhang, 2005)].

qPCR Primer:-

FOXO1-pro ChiP:- Forward 5’-ctgctgctccgtagtaaacaaag-3’
FOXO1-pro ChiP:- Reverse 5’-tctctttcacacactcacctcc-3’

2. BIM gene promoter (Human)

```
                                                                  FOXP3
-820 CTCGTTTTTT TTTTCCTAAA AGGATTTTTT TTTCTGAGTG CTTTTTTCAT TTCCCTATTT
-760 TACCTGCTTT GCACATGCCT CCCGCCCTCA CCCGGGAGGC GGCGAAGACG CCCACTGGGA
-700 CCGCGCCTGG CCGTTTTCTG GGCGCGTCCT GCCCCCACGG CCTCTGTCTC TTAGGGCGAC
-640 TGGGCGCGGA AGAAAAGCTG GAGAGCCCCT GCGGGGTGGC AGGAGGAGGG TGCCTGAGTC
-580 CCGCGAGAGG CCCGGGCGAG GAAGATGCGC AGCCTGCTGA TCCGCGTCCC GCCCGGCGCC
-520 AGGGACCCTC AGAGGGAGGA GAGCTCAAAG ACCTCGCCCC GCGCCTTCGC GAGGACCAAC
-460 CCAGTCCCCG CGCCTGCCCC AGAGCGGCTT AGAAACTCAG GGCACAGTGA GAGCGCAGGG
-400 CGCCTCCCGA GGCTTCACAC CGCCGGCCGG GCCAGCGGAG GCTGTGACCG GAGCGCCCCC
-340 TCTGTGCCGC GCACGCCGCG GCCGCCGCCG GGTTGGGGTA GGTGAGCGGG AGGCTAGGGT
                                                                     FOXO1
-280 ACACTTCGGG GTGGGGGATG GGCGCGCACA TGGCCGCCAG CAGGCAGAGT TACTCCGGTA
-220 AACACGCCAG GGACGGCGGC GCGCGCGGGA GGCAACCCAG CGGGGCAACC CCCGCCTTTA
-160 CCTGTCCGAG CCTGCACGCG CCGGCGGCCG CGGCGCCGAG GGGGGCGGAG CTTGCCAGCC
-100 CGCGCGCCGG GGCGGGACCT AGCGGGGGCG GGGTCCGCAG TGATTGGGCG TAGGAGCGGG

                                            ↱INS
 -40 GCCGCCAGCC AGAGCTGGGC TGCAGGGCCG CGCAGGTTTC acttcgctcc gcgcagccgc
 +21 ctggtctgca gtttgttgga gctctgcgtc cagcgccgct gccgctgccg ctgccgccgc
```

qPCR Primer:-

BIM-pro ChiP:- Forward 5’-gcttagaaactcagggcaca-3’
BIM-pro ChiP:- Reverse 5’-ccggagtaactctgcctgct-3’

3. FOXP3 gene promoter(human)

```
   1 ATCACTTGTG AGACATGTTC AGCACTGTAT CTGACCCATG GAAAATTCAA GATAAACATC
  61 AGCTACTGAG ATGATGGCGG ATATTTGGAA TCCTAAATCC TTGGAAACTG GGGCTTTTTG
 121 AAGTAAAAGA CCCCAAAGGC TGAGGGCCTC AGAAGCATCA GGCCATGATG TTCCTGAAAC
 181 AAGAGGGTCA GGGTCCCAAT GGGCCTCTGG GGTTCATCGT GAGGATGGAT GCATTAATAT
 241 TGGGGACCTG CTAGGGACCT TCCCAGTGGG ACAGTGGCTG GGTCAGGGCA CTCAAGCCCT
 301 AAAACGTGAT GAGGCGAGAC TTTTCTCTCT TTCCTCATTC AGTAACTGTC AGTAGATTCT
                                         FOXP3
 361 GGGAGCCAGG GATTCTCCGA CTCTTCAAGT CCATGAATTT TAGGGGATGA CAGTGGGCTC
 421 TCCGCTTTCT CCTCCATGAA GTAACTTACA TGCCCCTCAC CCTCTGTGGG AGGGGTGTTG
 481 CAGGGGGTGC AGAACTCCCC TCGCCGGGTA GTTCAAGCAA TGGGGACCAT ATCAATTCCA
 541 TCTATAGGGA AACTGAGGCC TGGAGTAGGG CGAGGCCTCT GGGAACCCAG CCCTATTCTG
 601 TCTCTTTCCC TGGCATTTCC CATCCACACA TAGAGCTTCA GATTCTCTTT CTTTCCCCAG
 661 AGACCCTCAA ATATCCTCTC ACTCACAGAA TGGTGTCTCT GCCTGCCTCG GGTTGGCCCT
           FOXO1
 721 GTGATTTATT TTAGTTCTTT TCCCTTGTTT TTTTTTTTTC AAACTCTATA CACTTTTGTT
 781 TTAAAAACTG TGGTTTCTCA TGAGCCCTAT TATCTCATTG ATACCTCTCA CCTCTGTGGT
                  FOXP3                                            FOXP3
 841 GAGGGGAAGA AATCATATTT TCAGATGACT CGTAAAGGGC AAAGAAAAAA ACCCAAAATT
 901 TCAAAATTTC CGTTTAAGTC TCATAATCAA GAAAAGGAGA AACACAGAGA GAGAGAAAAA
                                                    ↱INS
 961 AAAAACTATG AGAACCCCCC CCCACCCCGT GATTATCAGC gcacacactc atcgaaaaaa
1021 atttggatta ttagaagaga gaggtctgcg gcttccacac cgtacagcgt ggtttttctt
```

qPCR primer:-

FOXP3-pro ChiP:- Forward 5’-atggggaccatatcaattcc-3’
FOXP3-pro ChiP:- Reverse 5’-tttgccctttacgagtcatc-3’

Supplementary Figures (Original western blot)

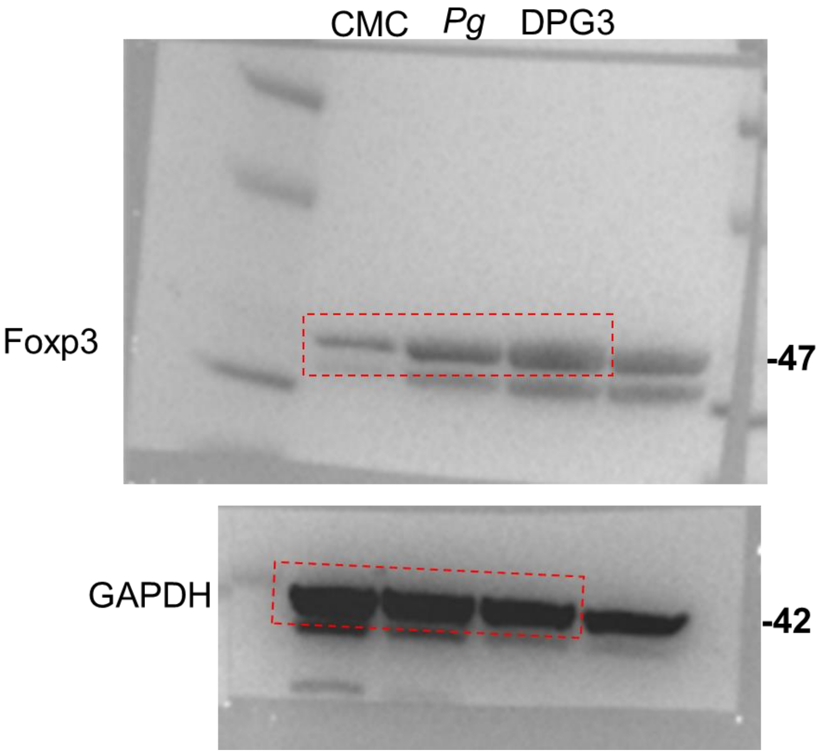

Supplementary Figure 1H. Original western blot to the corresponding figures (mice spleen tissue). Molecular weight markers are indicated in kDa.

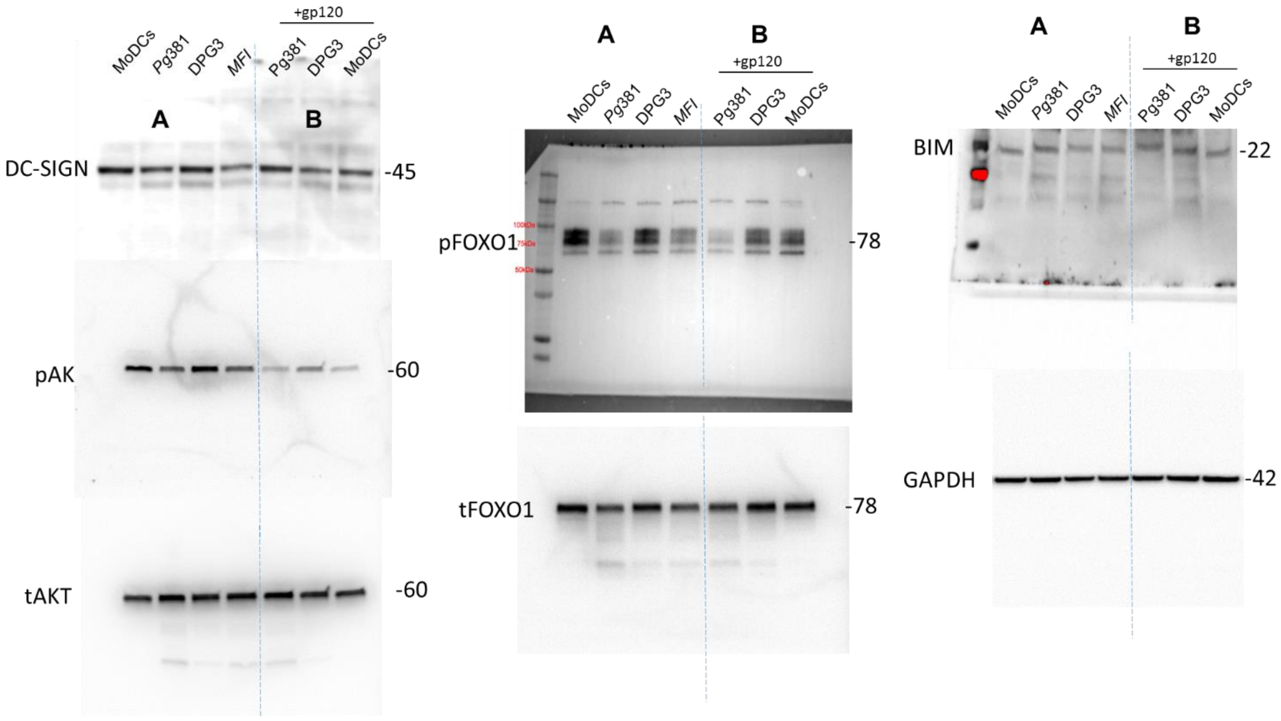

Supplementary Figure 2A, B. Original western blot to the corresponding figures (MDDSCs). Molecular weight markers are indicated in kDa.

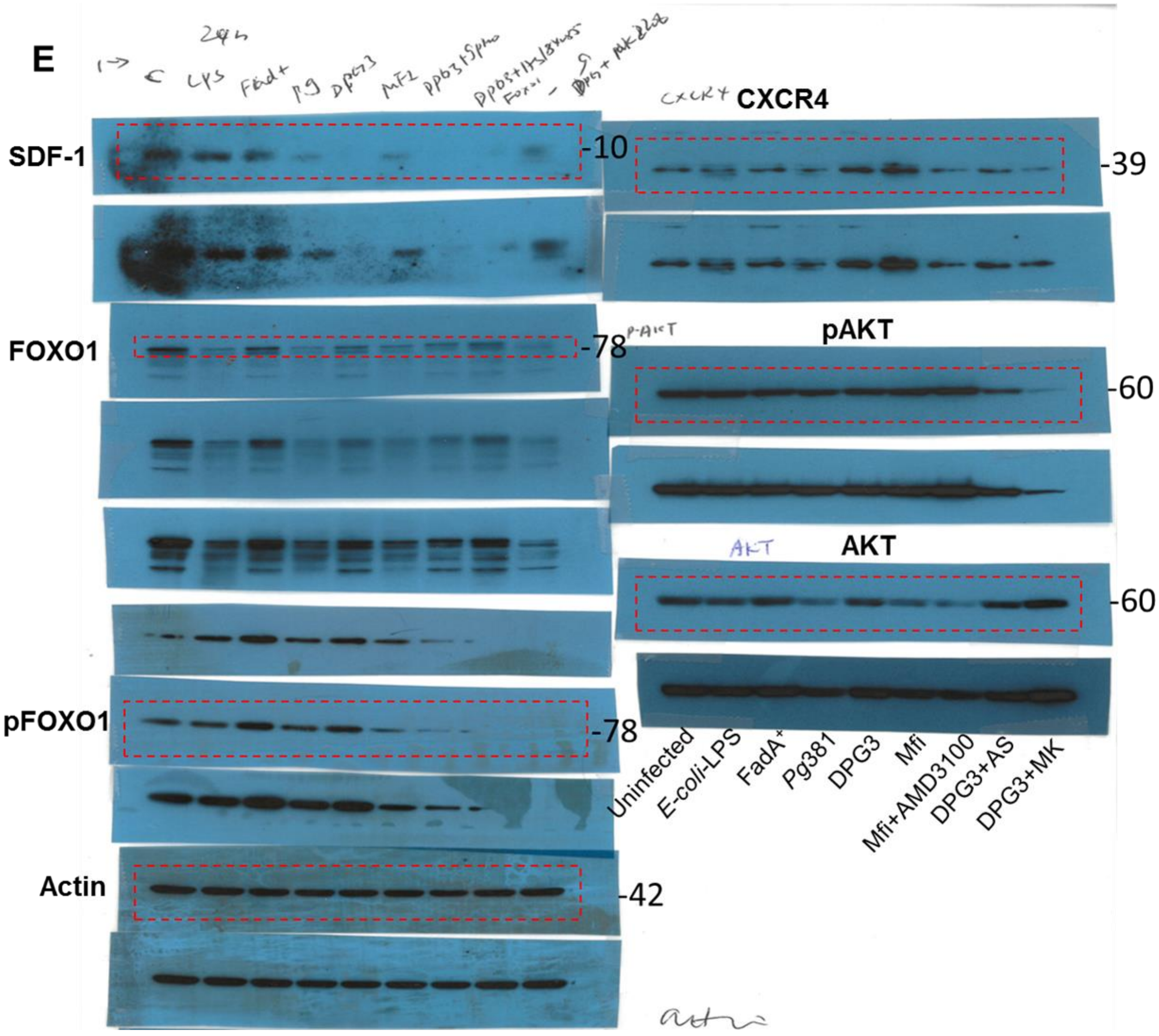

Supplementary Figure 3E. Original western blot films to the corresponding figures (Human head and neck oral squamous carcinoma cell (OSCCs) lines HN6). Molecular weight markers are indicated in kDa.

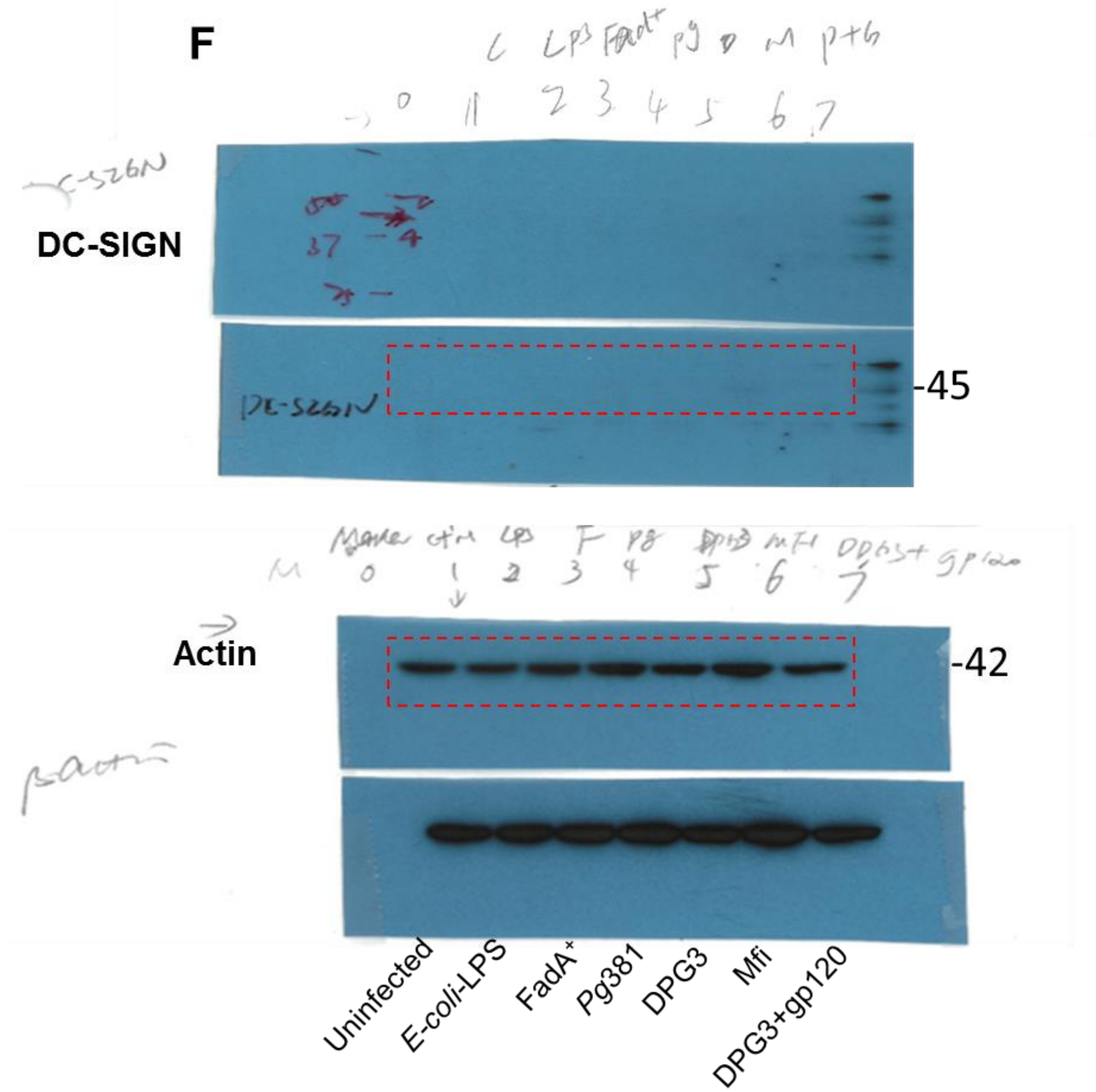

Supplementary Figure 3F. Original western blot films to the corresponding figures (Human head and neck oral squamous carcinoma cell (OSCCs) lines HN6). Molecular weight markers are indicated in kDa.

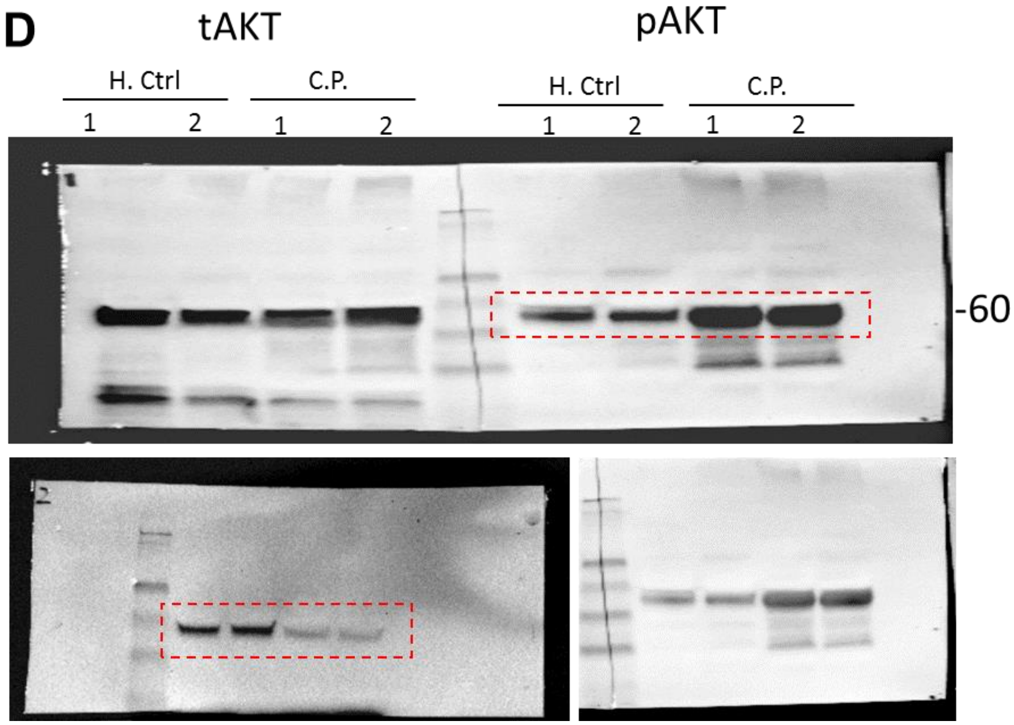

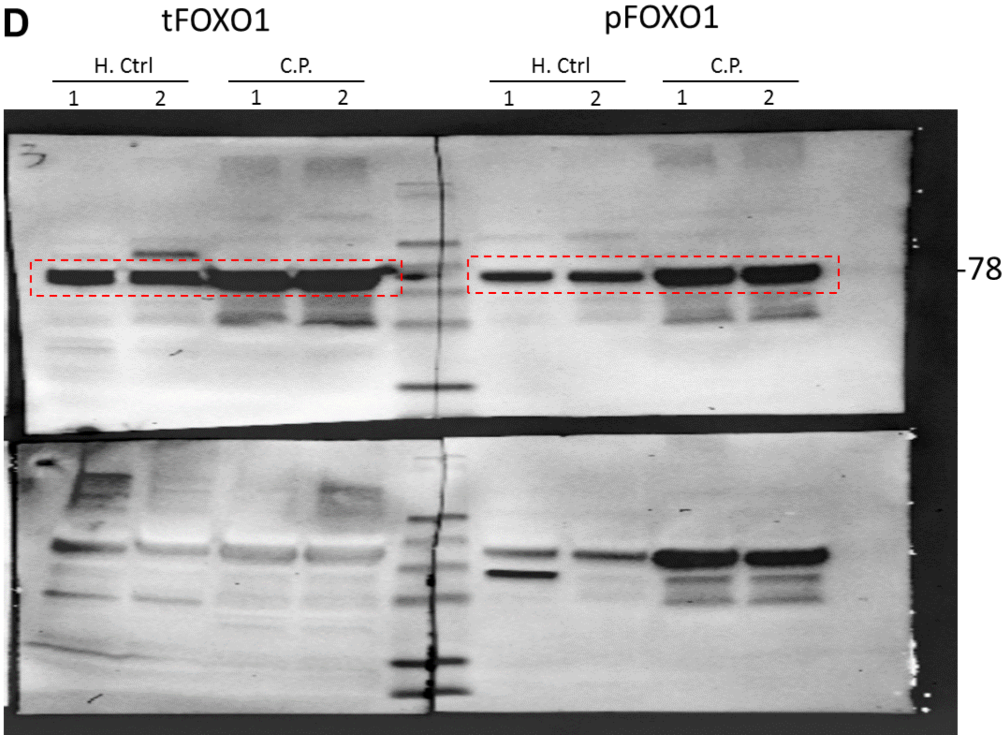

Supplementary Figure 4D. Original western blot to the corresponding figures (Human healthy control and CP gingival tissues). Molecular weight markers are indicated in kDa.

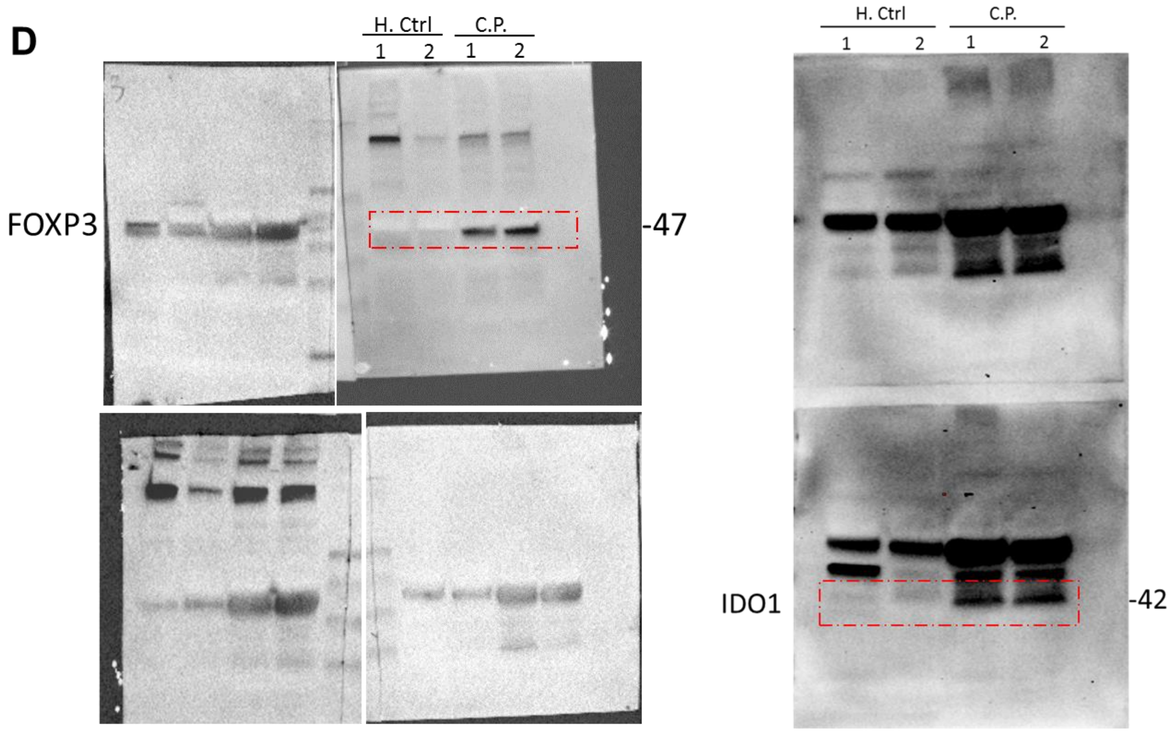

Supplementary Figure 4D. Original western blot to the corresponding figures (Human healthy control and CP gingival tissues). Molecular weight markers are indicated in kDa.

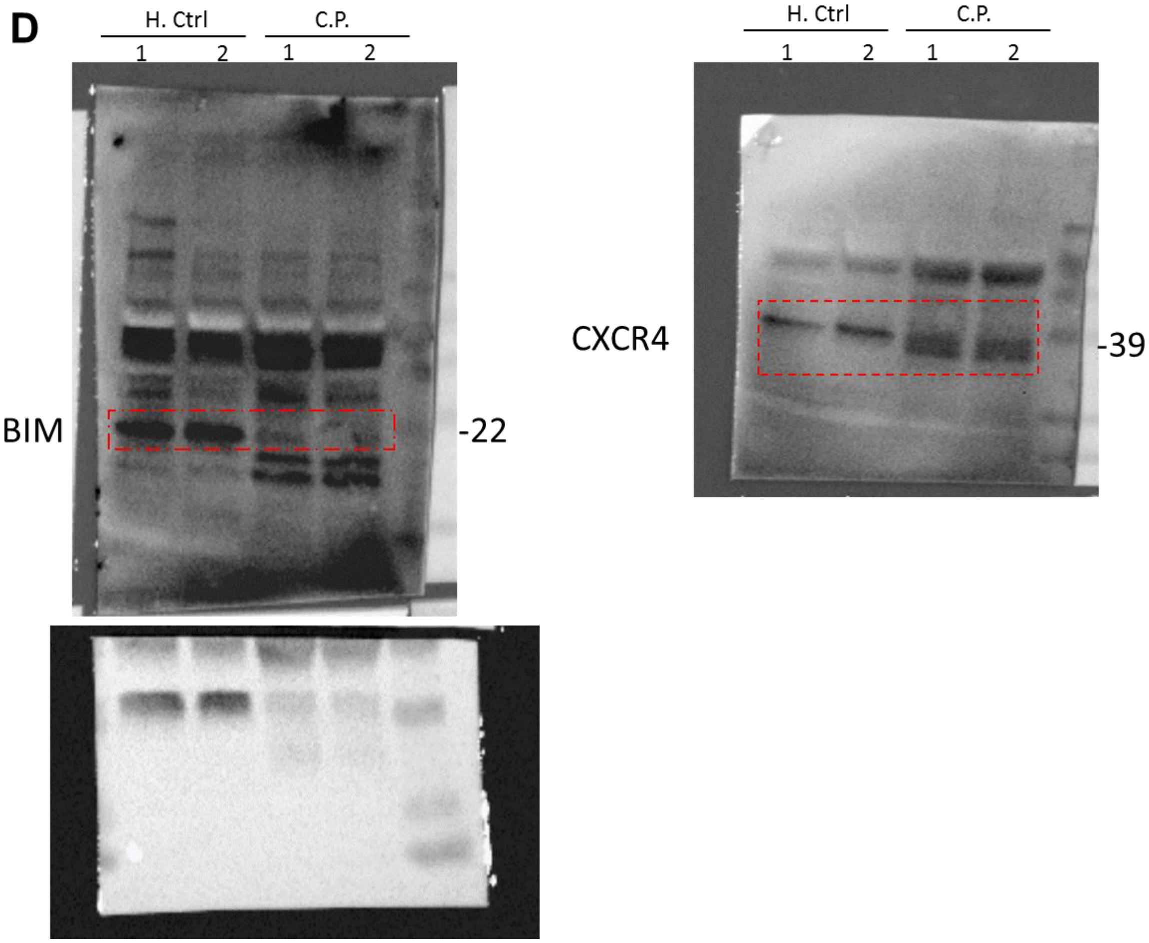

Supplementary Figure 4D. Original western blot to the corresponding figures (Human healthy control and CP gingival tissues). Molecular weight markers are indicated in kDa.

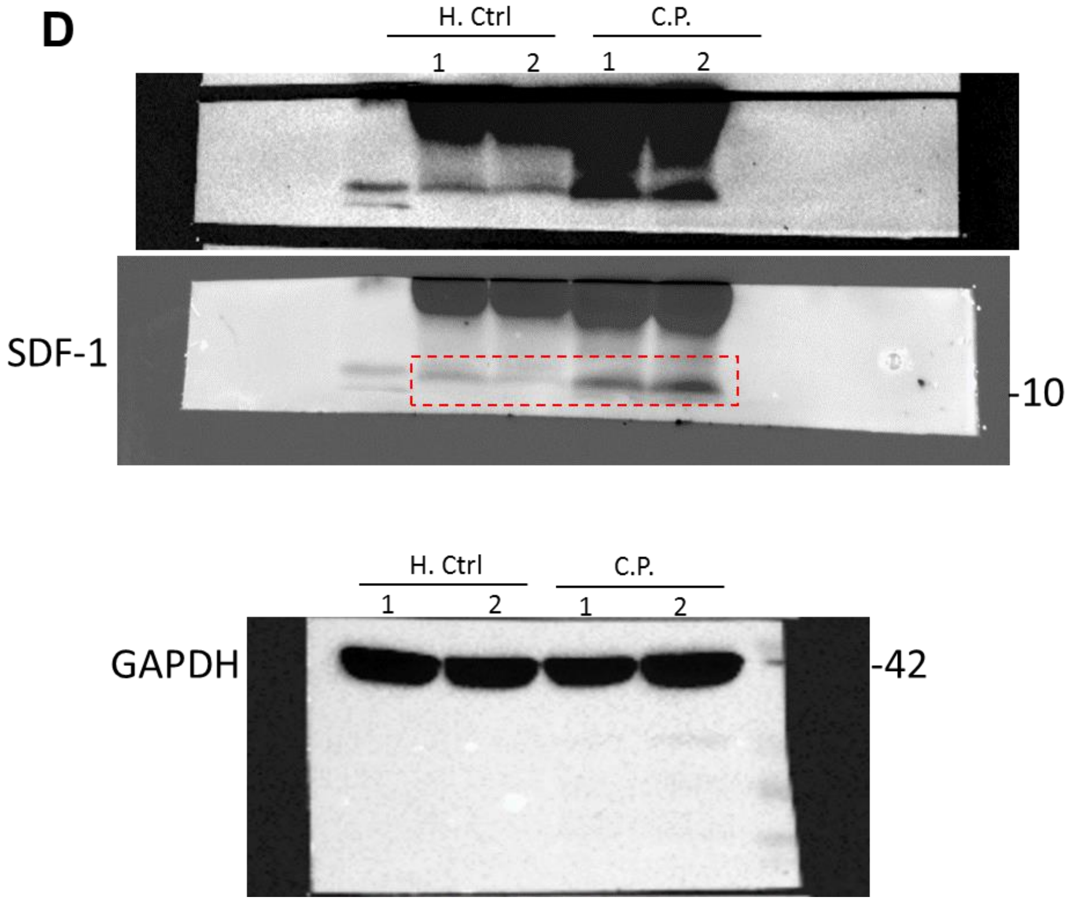

Supplementary Figure 4D. Original western blot to the corresponding figures (Human healthy control and CP gingival tissues). Molecular weight markers are indicated in kDa.
